# Quercetin Reduces Vascular Senescence and Inflammation in Symptomatic Male but Not Female Coronary Artery Disease Patients

**DOI:** 10.1111/acel.70108

**Published:** 2025-05-15

**Authors:** Pauline Mury, Olina Dagher, Annik Fortier, Ariel Diaz, Yoan Lamarche, Pierre‐Emmanuel Noly, Marina Ibrahim, Pierre Pagé, Philippe Demers, Denis Bouchard, Pierre‐Luc Bernier, Nancy Poirier, Emmanuel Moss, Nicolas Durrleman, Hughes Jeanmart, Michel Pellerin, Guillaume Lettre, Nathalie Thorin‐Trescases, Michel Carrier, Eric Thorin

**Affiliations:** ^1^ Montreal Heart Institute, Research Center Université de Montréal Montréal Québec Canada; ^2^ Department of Pharmacology & Physiology, Faculty of Medicine Université de Montréal Montréal Québec Canada; ^3^ Department of Cardiac Sciences Libin Cardiovascular Institute Calgary Alberta Canada; ^4^ Department of Biostatistics Montreal Health Innovations Coordinating Centre (MHICC) Montréal Québec Canada; ^5^ CIUSSS‐MCQ Université de Montréal, Campus Mauricie Trois‐Rivières Québec Canada; ^6^ Faculty of Medicine, Department of Surgery Université de Montréal Montréal Québec Canada; ^7^ Department of Medicine, Faculty of Medicine Université de Montréal Montréal Québec Canada

**Keywords:** cellular senescence, coronary artery disease, endothelium‐dependent relaxation, *inflammaging*, sex‐dimorphism, snRNA‐seq, systemic inflammatory proteomic

## Abstract

Recent studies suggest that vascular senescence and its associated inflammation fuel the *inflammaging* to favor atherogenesis; whether these pathways can be therapeutically targeted in coronary artery disease (CAD) patients remains unknown. In a randomized, double‐blind trial, 97 patients (78 men) undergoing coronary artery bypass graft surgery were treated with either quercetin (500 mg twice daily, 47 patients) or placebo (50 patients) for two days pre‐surgery through hospital discharge. Primary outcomes were reduced inflammation and improved endothelial function ex vivo. Exploratory analyses included plasma proteomics and single‐nuclei RNA sequencing of internal thoracic artery (ITA) samples. Quercetin treatment showed a trend toward reduced C‐reactive protein at discharge (*p* = 0.073) and differentially modulated circulating inflammatory protein expression between men and women, with a pro‐inflammatory effect of quercetin in females. Endothelial acetylcholine‐induced relaxation improved significantly with quercetin (*p* = 0.049), with effects in men (*p* = 0.043) but not in women (*p* = 0.852). ITA transcriptomics revealed the overexpression of senescence and *inflammaging* pathways in male vascular cells, which quercetin reversed. In female cells, quercetin had minimal endothelial benefit and increased *inflammaging* in fibroblasts. In male cells, a candidate target of quercetin involves interactions between the receptor *PLAUR* and its ligands *PLAU* and *SERPINE1*. Post‐operative atrial fibrillation incidence was significantly lower with quercetin, representing 4% of the patients compared to 18% in the placebo group (*p* = 0.033). In conclusion, short‐term quercetin treatment effectively targeted vascular senescence in male CAD patients, improving inflammatory and functional outcomes. However, these benefits were not observed in female patients.

**Trial Registration:** https://clinicaltrials.gov, NCT04907253

## Graphical abstract

In male patients undergoing CABG surgery, pretreatment with quercetin suppressed *inflammaging* and its associated inflammation, leading to improved endothelium‐dependent relaxation (measured ex vivo) and reducing new onset of post‐operative atrial fibrillation. These benefits were not observed in female patients, who exhibited minimal evidence of *inflammaging*.

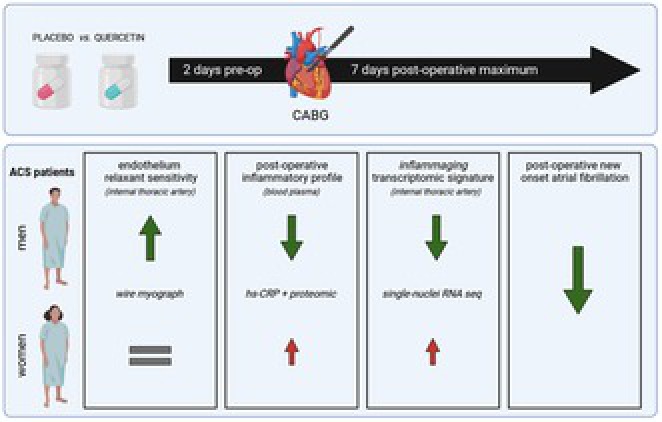

## 1 Introduction

Numerous patients on therapies addressing modifiable risk factors for cardiovascular diseases (CVD) remain at risk of cardiovascular complications, demonstrating that residual risk is not entirely addressed. In 2000, the concept of *inflammaging* was proposed (Franceschi et al. 2000), postulating that with age, and in synergism with risk factors for CVD, cellular damages promote the accumulation of senescent cells, feeding inflammation (Liberale et al. 2022). Indeed, while at irreversible cell cycle arrest, senescent cells remain metabolically active (McHugh and Gil 2018) and release numerous proinflammatory factors, proteases, and insoluble proteins/extracellular matrix components, gathered into the Senescence‐Associated Secretory Phenotype (SASP) (Coppé et al. 2010; Kuilman et al. 2010). The SASP is potentially harmful when senescent cells accumulate by generating a chronic low‐grade inflammatory environment (Childs et al. 2017) common to all age‐related chronic diseases (Liberale et al. 2022). It is now demonstrated that senescent cells beget senescence and that this effect is dependent on the SASP (Kirkland et al. 2017; Xu et al. 2018).

Elements of the SASP can be antagonized to reduce the damaging effects of senescent cells by drugs called senomorphic or senostatic (Childs et al. 2017), i.e., drugs that do not kill senescent cells but reduce the detrimental effects of the SASP by targeting the residual inflammation (Kirkland et al. 2017). Data from the CANTOS trial showed that neutralizing IL‐1β, a prominent SASP member (Malaquin et al. 2016), with canakinumab benefited patients with coronary artery disease (CAD) (Ridker et al. 2017, 2018). However, side effects were reported, highlighting that new selective therapeutic targets for vascular senescent cells are desirable. Senolytics, i.e., drugs that kill senescent cells, are other potential candidates. The current drug candidates arise from the oncology therapeutic armamentarium and include inhibitors of the Bcl2 family of anti‐apoptotic proteins and tyrosine kinases; used chronically, these drugs generate side effects (Caldemeyer et al. 2016; Wilson et al. 2010). However, treatment can be intermittent: sequential short‐term treatment of old mice with dasatinib combined with quercetin (tyrosine kinase inhibitor/multiple targets) killed senescent cells by apoptosis and improved endothelial cells (EC) relaxant function in aged and in atherosclerotic mice (Roos et al. 2016). ABT‐263 (navitoclax, a Bcl2 inhibitor) reduced atherosclerosis in mice (9‐day treatment) (Childs et al. 2016). Less than a decade ago, Kirkland's group reported that quercetin alone had senolytic activity on senescent but not on proliferating human EC (Zhu et al. 2017, 2015). Because of the high safety profile of quercetin (Dagher, Mury, Thorin‐Trescases, et al. 2021), it makes this natural product an ideal candidate to test whether intermittent endothelial senolysis would decrease senescent cellular load in the arterial wall of patients, reduce the *inflammaging* transcriptomic signature and improve endothelial function, a strong predictor of future cardiovascular adverse events (Halcox et al. 2002; Shechter et al. 2009). Although senolytics have been clinically tested in different pilot studies, such as in diabetic patients with kidney disease (Hickson et al. 2019) and in mild Alzheimer disease patients (Gonzales et al. 2023), to the best of our knowledge, the effect of senolytics has never been studied in CAD patients.

We designed this clinical trial to test the hypothesis that a short‐term quercetin treatment would reduce activation of senescent pathways and its associated *inflammaging* signature in EC of lesion‐free arteries from CAD patients (Dagher, Mury, Noly, et al. 2021). Therefore, we performed a randomized, double‐blind trial involving male and female adults undergoing inpatient coronary artery bypass graft (CABG) surgery following a recent acute coronary syndrome (ACS). Briefly, the primary endpoints were a reduction in post‐operative systemic inflammation and improved endothelial function ex vivo of discarded segments of the internal thoracic artery (ITA) (Dagher, Mury, Noly, et al. 2021). The exploratory endpoints were to acquire a plasma proteomics profile and a vascular (ITA) transcriptomic signature by single‐nucleus RNA sequencing (Dagher, Mury, Noly, et al. 2021). In light of our recent data revealing a strong sexual dimorphism in the endothelial *inflammaging* signature of these patients (Mury et al. 2024), we also aimed to investigate whether the efficacy of quercetin would be sex‐dependent.

## 2 Methods

### 2.1 Study Design and Population

Q‐CABG (ClinicalTrials.gov, NCT04907253) was a prospective, multi‐centre, randomized, double‐blind, allocation‐concealed, and placebo‐controlled study involving patients of both sexes undergoing an elective CABG surgery. The trial protocol was approved by each local institutional review board and by Health Canada and was conducted in accordance with the principles of Good Clinical Practice, and the trial conformed to the principles outlined in the Declaration of Helsinki. Its design and rationale have been published previously (Dagher, Mury, Noly, et al. 2021). In brief, the effect of quercetin treatment was tested on hs‐CRP profile (before surgery and up to hospital discharge at postoperative day (POD) 7), on the systemic proteomic inflammatory signature at POD4 of 384 markers (Olink), on endothelial relaxant function ex vivo using acetylcholine (ACh, day of the surgery), and on the transcriptomic signature using single‐nucleus RNA sequencing (snRNA‐seq, day of the surgery). All measures were obtained from the intent‐to‐treat (ITT; all randomized patients were included in the final analysis, even if they deviated from the protocol) patient population, except for the snRNA‐seq that was performed in 12 patients *per* protocol (no deviation from the protocol) blindly selected from this population of patients.

Patients ≥ 18 years of age who experienced a recent ACS within the previous month, including a diagnosis of unstable angina, non‐ST elevation myocardial infarction (NSTEMI) or ST‐elevation myocardial infarction (STEMI), and who were scheduled to undergo an inpatient CABG surgery at the Montreal Heart Institute were approached for the study (June 2021 to June 2023). A patient was ineligible if they required a combined procedure (e.g., a combined CABG and valve intervention), had documented severe kidney or liver failure, were on immunosuppressive therapy, or if they were pregnant or breastfeeding. Complete inclusion and exclusion criteria are outlined in Table S1.

### 2.2 Interventions

After obtaining written consent, patients were randomly assigned in a 1:1 ratio to either the intervention arm, in which they received quercetin supplementation (500 mg *per os* bid), or the control arm, in which they received a placebo. The randomization list was computer‐generated by an unblinded biostatistician from the Montreal Health Innovations Coordinating Center (MHICC). The trial medication and matching placebo were provided free of charge by Advanced Orthomolecular Research (AOR) Inc. (Calgary, Alberta, Canada); AOR had no role in the design or conduct of the trial.

Quercetin or placebo administration was started 2 days before planned surgery and was continued after surgery until hospital discharge or for a maximum of seven POD. Hospital stay was not prolonged for the sole purpose of the study. All participants were managed by their primary care team and were treated according to national guidelines that included optimal medical therapy for the treatment of acute myocardial infarctions. The baseline demographic and clinical characteristics of the participants in the treatment arms are shown in Table 1.

**TABLE 1:** General characteristics of the intent‐to‐treat cohort at baseline (pre‐treatment).

	All patients	Quercetin	Placebo	*p*
	(*n* = 97)	(*n* = 47)	(*n* = 50)	
*Cardiovascular risk factors*				
Age (years)	67.0 ± 1.0	68.5 ± 1.2	65.6 ± 1.5	0.14
Sex				
Men	78 (80.4%)	39 (83.0%)	39 (78.0%)	0.54
Women	19 (19.6%)	8 (17.0%)	11 (22.0%)	
BMI (kg/m^2^)	29.3 ± 0.5	29.3 ± 0.7	29.3 ± 0.8	0.95
Hypertension	77 (79.4%)	37 (78.7%)	40 (80.0%)	0.88
Dyslipidemia	90 (92.8%)	44 (93.6%)	46 (92.0%)	> 0.99
Diabetes mellitus	38 (39.2%)	19 (40.4%)	19 (38.0%)	0.81
COPD	11 (11.3%)	6 (12.8%)	5 (10.0%)	0.67
Obstructive sleep apnea	14 (14.4%)	5 (10.6%)	9 (18.0%)	0.30
Previous stroke	5 (5.1%)	2 (4.2%)	3 (6.0%)	> 0.99
Previous TIA	3 (3.1%)	2 (4.2%)	1 (2.0%)	> 0.99
Peripheral vascular disease	10 (10.3%)	7 (14.9%)	3 (6.0%)	0.15
Smoking				
Active	19 (19.6%)	11 (23.4%)	8 (16.0%)	0.36
Ex‐smoker	42 (43.3%)	19 (40.4%)	23 (46.0%)	0.58
Previous MI	17 (17.5%)	10 (21.3%)	7 (14.0%)	0.35
Previous PCI	18 (18.6%)	10 (21.3%)	8 (16.0%)	0.50
Chronic kidney disease	13 (13.4%)	9 (19.1%)	4 (8.0%)	0.11
Known AF	6 (6.2%)	5 (10.6%)	1 (2.0%)	0.10
Alcohol abuse	6 (6.2%)	4 (8.5%)	2 (4.0%)	0.36
Illicit drug use	1 (1.0%)	1 (2.1%)	0 (0.0%)	0.48
*Home medication*				
Aspirin	60 (61.8%)	24 (51.1%)	36 (72.0%)	0.03*
Other anti‐platelet agent	9 (9.3%)	4 (8.5%)	5 (10.0%)	0.80
Warfarin	1 (1.0%)	1 (2.1%)	0 (0.0%)	0.30
DOAC	5 (5.1%)	5 (10.6%)	0 (0.0%)	0.02*
Statin	73 (75.2%)	33 (70.2%)	40 (80.0%)	0.26
Beta blocker	37 (38.1%)	19 (40.4%)	18 (36.0%)	0.65
ACEI	27 (27.8%)	13 (27.7%)	14 (28.0%)	0.97
ARA II	21 (21.6%)	9 (19.2%)	12 (24.0%)	0.56
Hypoglycemic agent	34 (35.0%)	17 (36.2%)	17 (34.0%)	0.82
SGLT2i	10 (10.3%)	3 (6.4%)	7 (14.0%)	0.22
DPP4	7 (7.2%)	4 (8.5%)	3 (6.0%)	0.63
Insulin	4 (4.1%)	2 (4.2%)	2 (4.0%)	0.95
Gabapentin	7 (7.2%)	6 (12.8%)	1 (2.0%)	0.04*
HRT	2 (2.0%)	1 (2.1%)	1 (2.0%)	0.96
NSAID	1 (1.0%)	1 (2.1%)	0 (0.0%)	0.30
*Clinical presentation*				
Unstable angina	48 (49.5%)	23 (48.9%)	25 (50.0%)	0.70
NSTEMI	41 (42.3%)	19 (40.4%)	22 (44.0%)	
STEMI	8 (8.2%)	5 (10.6%)	3 (6.0%)	
Preoperative IABP	3 (3.1%)	0 (0.0%)	3 (6.0%)	0.09
LVEF, %	50.1 ± 1.1	48.6 ± 2.0	51.5 ± 1.2	0.83
RV dysfunction	7 (7.2%)	6 (12.8%)	1 (2.0%)	0.06
*Biochemistry*				
hs‐CRP (mg/L)	7.3 ± 1.5	7.6 ± 2.5	7.1 ± 1.8	0.48
LDL cholesterol	1.93 ± 0.11	1.94 ± 0.17	1.91 ± 0.15	0.99
HDL cholesterol	1.16 ± 0.03	1.15 ± 0.05	1.17 ± 0.04	0.99
HbA1c, %	6.25 ± 0.14	6.31 ± 0.22	6.19 ± 0.19	0.51
Creatinine	82.3 ± 2.0	86.1 ± 3.2	78.7 ± 2.2	0.06
Hemoglobin	137 ± 1.6	139 ± 2.2	136 ± 2.3	0.18
*Surgical profile*				
Syntax score	20.5 ± 0.9	20.1 ± 1.2	20.8 ± 1.3	0.76
STS score, %	1.11 ± 0.09	1.29 ± 0.15	0.92 ± 0.10	0.10
Euroscore II, %	1.88 ± 0.3	2.49 ± 0.5	1.18 ± 0.1	0.44
Off‐pump	20 (20.6%)	13 (27.7%)	7 (14.0%)	0.09
CPB time (min)	82.6 ± 3.16	79.7 ± 4.26	85.8 ± 4.69	0.39
Aortic clamp duration (min)	64.5 ± 2.57	62.2 ± 3.81	67.0 ± 3.46	0.41
Blood cardioplegia	29 (29.9%)	14 (29.8%)	15 (30.0%)	0.68
Del Nido cardioplegia	47 (48.4%)	25 (53.2%)	22 (44.0%)	
Number of distal anastomoses				
1	6 (6.2%)	3 (6.4%)	3 (6.0%)	0.65
2	16 (16.5%)	7 (14.9%)	9 (18.0%)	
3	49 (50.5%)	27 (57.5%)	22 (44.0%)	
4	20 (20.6%)	7 (14.9%)	13 (26.0%)	
5	6 (6.2%)	3 (6.4%)	3 (6.0%)	
Skeletonized ITA harvesting	62 (63.9%)	31 (65.9%)	31 (62.0%)	0.68

*Note:* Values are expressed as mean ± SEM, and percent within the group. Statistical analyses comparing Placebo and Quercetin groups were performed using ANOVA for continuous variables and a chi‐square for categorial variables. *p < 0.05 vs. placebo.

Abbreviations: ACEI, angiotensin converting enzyme inhibitor; AF, atrial fibrillation; ARA, Angiotensin II receptor antagonist; BMI, body mass index; CBP, cardiopulmonary bypass; COPD, chronic obstructive pulmonary disease; DOAC, direct oral anticoagulant; DPP4, dipeptidyl peptidase 4; HbA1c, hemoglobin A1c; HRT, hormone replacement therapy; IABP, intra‐aortic balloon pump; ITA, internal thoracic artery; LVEF, left ventricular ejection fraction; MI, myocardial infarction; NSAID, non‐steroidal anti‐inflammatory drug; NSTEMI, non‐ST elevation myocardial infarction; PCI, percutaneous coronary intervention; RV, right ventricular; SGLT2i, sodium‐glucose cotransporter‐2 inhibitor; STEMI, ST elevation myocardial infarction; STS, society of thoracic surgeons; TIA, transient ischemic attack.

### 2.3 Follow‐Up

Quercetin is a very safe substance (Dagher, Mury, Thorin‐Trescases, et al. 2021). Regardless, tolerance of the trial therapy was still assessed continuously during patients' hospital stay. No allergic reaction or serious adverse effects were attributed to the trial drug. Seven patients independently withdrew their consent during the study (Figure S1). As mentioned above, the trial therapy was stopped at hospital discharge. Participants were contacted over the phone by a member of the research team after one month. Information about their post‐operative recovery, including symptoms or complications (wound infection, recurrent angina, recurrent hospitalization, atrial fibrillation, stroke, etc.) was collected (Table 2). Post‐operative new onset events occurring during hospitalization and *de novo* events developing during the 30‐day follow‐up correspond to cardiovascular events observed in patients who had no such event prior to surgery or before hospital discharge (Table 2).

**TABLE 2:** Post‐operative and follow‐up characteristics of the intent‐to‐treat cohort.

	All patients	Quercetin	Placebo	*p*
	(*n* = 97)	(*n* = 47)	(*n* = 50)	
Post‐operative consequences				
Vasoplegia	18 (18.5%)	10 (21.3%)	8 (16.0%)	0.47
New onset Atrial fibrillation	11 (11.3%)	2 (4.2%)	9 (18.0%)	0.03*
Infection	4 (4.1%)	1 (2.1%)	3 (6.0%)	0.62
Stroke	2 (2.0%)	2 (4.2%)	0 (0.0%)	0.23
New onset kidney disease	2 (2.0%)	2 (4.2%)	0 (0.0%)	0.23
Colchicine	5 (5.1%)	3 (6.4%)	2 (4.0%)	0.67
NSAID	76 (78.3%)	34 (72.3%)	42 (84.0%)	0.16
Hs‐CRP at POD4 (mg/L)	121.2 ± 6.9	125.3 ± 7.0	117.1 ± 6.9	0.41
30‐day follow‐up				
Survival	97 (100%)	47 (100%)	50 (100%)	—
Recurrent angina	0 (0.0%)	0 (0.0%)	0 (0.0%)	—
Recurrent ACS	0 (0.0%)	0 (0.0%)	0 (0.0%)	—
*De novo* AF	1 (1.0%)	1 (2.1%)	0 (0.0%)	0.48
Hospital readmission	8 (8.2%)	4 (8.5%)	4 (8.0%)	> 0.99
Leg infection	7 (7.2%)	3 (6.4%)	4 (8.0%)	> 0.99
Sternal infection	3 (3.1%)	1 (2.1%)	2 (4.0%)	> 0.99
Graft failure	0 (0.0%)	0 (0.0%)	0 (0.0%)	—

*Note:* Values are expressed as mean ± SEM, and percent within the group. Statistical analyses comparing Placebo and Quercetin groups were performed using ANOVA for continuous variables and a chi‐square for categorial variables. *p < 0.05 vs. placebo.

Abbreviations: ACS, acute coronary syndrome; AF, atrial fibrillation; NSAID, non‐steroidal anti‐inflammatory drug.

### 2.4 Statistical Analysis

All statistical analyses were performed using SAS Version 9.4 or higher (SAS Institute Inc., Cary, NC, USA). All statistical tests were two‐tailed and a *p*‐value < 0.05 was considered statistically significant. No adjustment for multiple testing was done, and no missing data was imputed.

#### 2.4.1 Power Statistical Calculation

Power calculation was based on the data collected in a previous study (Noly et al. 2019) and did not consider sex as a variable: the standard deviation assumed for the ratio (hs‐CRP at POD4/hs‐CRP at baseline) log‐transformed (i.e., the standard deviation for log [(hs‐CRP at POD4)—log (hs‐CRP at baseline)]) to be common to both groups was 1.215. A sample of 50 subjects *per* group (100 subjects in total) would detect a relative reduction of 50% in the change in hs‐CRP in the quercetin group compared to the placebo group, with a two‐tailed significance level of 0.05 and a power of 80%. The complete design of the study has been published in (Dagher, Mury, Noly, et al. 2021).

#### 2.4.2 Analyses of Baseline Parameters

Continuous variables are shown as mean ± standard error of the mean (SEM) or median (25th and 75th percentiles). Groups were compared using Student's *t*‐test or Mann–Whitney‐Wilcoxon test for continuous variables. Categorical data are presented as absolute frequencies with percentages, and groups were compared using chi‐square or Fisher exact test.

#### 2.4.3 Analyses of Hs‐CRP Blood Levels

Changes from baseline to follow‐up in blood levels of hs‐CRP were compared between the two groups (quercetin or placebo) using a 2‐way repeated measures covariance analysis (ANCOVA), including the following terms: groups (quercetin or placebo), time points (POD1, POD4, POD7), an interaction term between the groups and time points, and a term for the baseline hs‐CRP value (collected the morning before the first dose of treatment, 2 days before the surgery). Whether the interaction term was statistically significant or not, contrasts were produced to compare groups on POD4 for the primary objective, and then for other indicated time points as exploratory analyses.

#### 2.4.4 Analyses of Olink Systemic Proteomic Inflammatory Signature

Proteomics analyses were performed using R software (v 4.3.2). Multiple linear regression analyses were performed using the Limma package (Ritchie et al. 2015).

#### 2.4.5 Analyses of EC_50_

Because of their non‐normal distribution, EC_50_ (the concentration of ACh that induces 50% of the maximal relaxation measured in each donor arterial segment) in placebo and quercetin groups was compared using the Mann–Whitney‐Wilcoxon test.

#### 2.4.6 Analysis of snRNA‐Seq Transcriptomic

The dataset was analyzed as previously described (Mury et al. 2024).

A detailed description of all methods and analyses is provided in the Supporting Information.

## 3 Results

### 3.1 Patient Characteristics

Between June 2021 and June 2023, 111 patients were enrolled and randomized to receive quercetin (*n* = 55) or placebo (*n* = 56). A total of 97 patients (the intent‐to treat (ITT) population) were finally analyzed (Figure S1). Of these 97 patients, 47 (48.4%) received quercetin (39 men and 8 women), and 50 (51.5%) received a placebo (39 men and 11 women); there was no cross‐over of treatment during the study. The mean age of the participants was 67.0 ± 1.0 years (66.6 ± 1.1 years for men and 68.8 ± 1.8 years for women, *p* = 0.39). The baseline demographic and clinical characteristics of the participants in the treatment arms are shown in Table 1; clinical characteristics divided by sex and treatment are in Table S2. All women were menopaused, and one female patient in both the placebo and quercetin groups received hormonal replacement therapy (Table 1). Fewer patients in the placebo group received aspirin, but more patients in the quercetin group received direct oral anticoagulant (Table 1). Forty‐four (44) segments of ITA in the placebo group and 34 segments in the quercetin group were made available by the operating surgeon and used for endothelial relaxant function, while 12 adjacent segments of ITA were used for the acquisition of a snRNA‐seq transcriptomic signature. For the two primary endpoints—inflammation and endothelial function ex vivo—data are first presented without considering sex as a variable, followed by a presentation of the data categorized by sex.

### 3.2 Post‐Operative Events and Follow‐Up

Post‐operative new onset CV events were followed until hospital discharge, and during a 4‐week follow‐up after hospital discharge (Table 2). No serious adverse effect of quercetin or placebo treatment was reported during the study. Remarkably, the incidence of new onset postoperative atrial fibrillation (AF) while patients were still hospitalized was significantly reduced by quercetin compared to placebo (quercetin: 4%, i.e., 2/47, vs. placebo: 18%, i.e., 9/50, *p* = 0.033; Table 2). In the placebo group, 8 men and 1 woman developed new onset AF, while only 2 men developed AF in the quercetin group (*χ* ^2^ test, *p* = 0.128). No confounders were identified, such as left atrium size, prior pericardiotomy, or AF prophylaxis peri‐ and post‐operatively, to impact new onset AF; no proteomic biomarkers were identified as predictors of new onset AF due to the low event rate. No effect of quercetin was observed at the 4‐week follow‐up, and there was no difference in the *de novo* development of AF during the follow‐up between groups (Table 2).

### 3.3 Effect of a Quercetin Treatment on Plasma Inflammatory Markers

**FIGURE 1:**
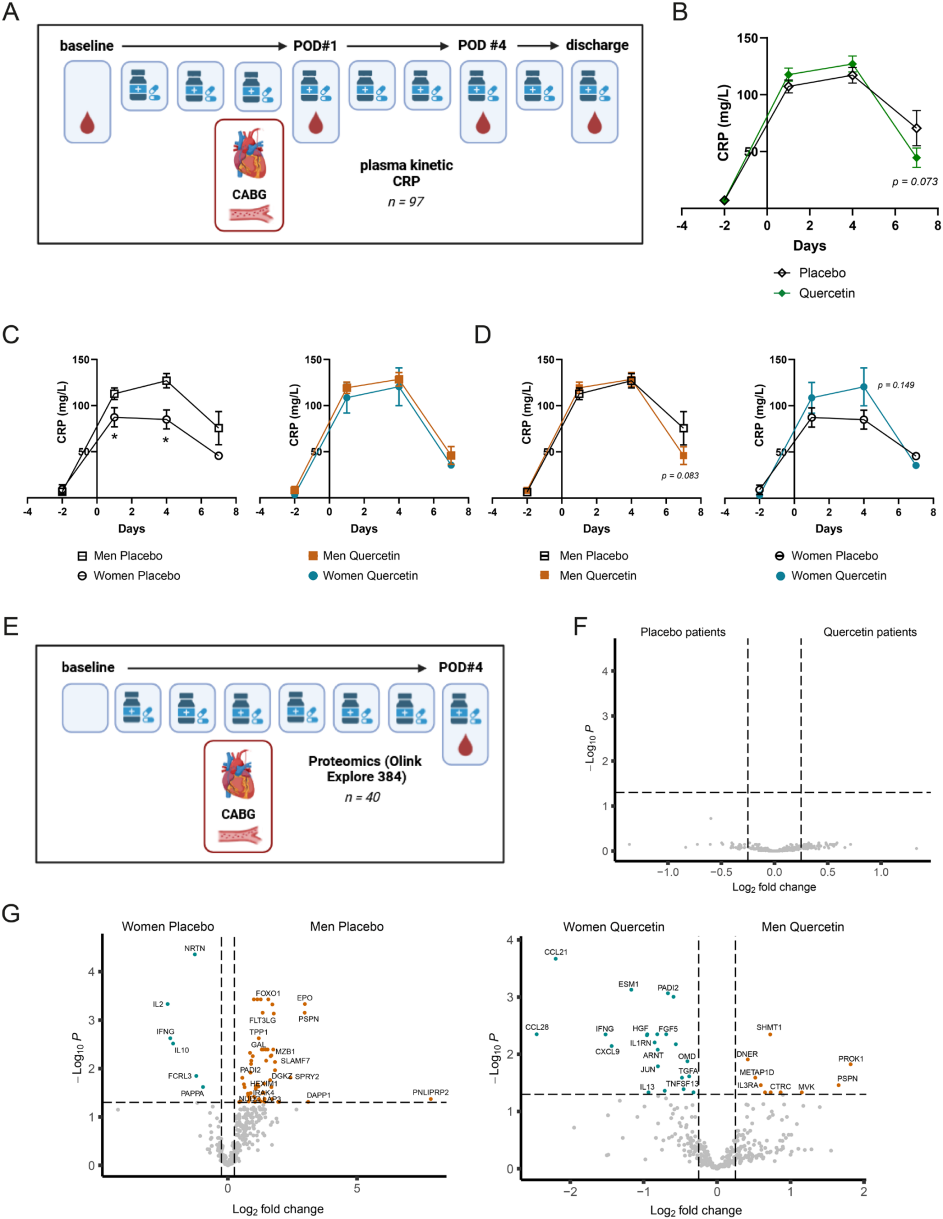
Impact of quercetin on systemic inflammatory biomarkers. (A) Flow‐chart of the primary endpoint of the study (impact of quercetin on systemic inflammation) at each timepoint of hs‐CRP measurement (created using BioRender). (B) Mean plasma hs‐CRP levels measured at baseline (pre‐treatment), post‐operative day (POD) 1, POD4 and at hospital discharge (POD7) after a coronary by‐pass graft surgery, in the intent‐to‐treat (ITT; 97 patients) population. Data are mean ± SEM of 50 placebo patients vs. 47 quercetin patients. ANCOVA was performed (effect of Group *p* = 0.824, Time *p* < 0.0001, Group × Time *p* = 0.025). (C) Mean plasma hs‐CRP levels of 39 men placebo patients vs. 11 women placebo patients and of 39 men quercetin patients vs. 8 women quercetin patients. *p < 0.05 vs. men placebo. (D) Mean plasma hs‐CRP levels of 39 men quercetin vs. 39 men placebo (left panel) and 8 women quercetin vs. 11 women placebo (right panel). (E) Flowchart of the proteomic analysis (Olink Proteomics) at POD4 (created using BioRender). (F) VolcanoPlot visualization of 384 inflammation‐related biomarkers in placebo vs. quercetin patients illustrating any differentially expressed proteins (DEP) between placebo and quercetin groups in the ITT population (proteins are considered significantly differently expressed when false discovery rate (FDR) < 0.05 and Log2 fold change (L2FC) > 0.25). (G) All DEP of the Inflammation panel between women placebo and men placebo groups (left), and comparison between women quercetin and men quercetin (right) (proteins are considered significantly differently expressed when FDR < 0.05 and L2FC > 0.25).

To assess the potential beneficial effect of quercetin on systemic inflammation, we used 2 different strategies (Figure 1): first, we assessed the temporal evolution of the inflammatory response (primary endpoint) of plasma hs‐CRP by a 4‐timepoint analysis (baseline, i.e., morning before the first dose given 2 days before the surgery, and at POD1, POD4 and POD7, i.e., after 9 days of quercetin) (Figure 1A–D); second, as an exploratory endpoint, we used the Olink Explore 384 Inflammation panel at POD4 (after 6 days of quercetin) in the plasma of 40 blindly and randomly selected patients (30 men and 10 women) to compare the global inflammatory signature between sexes only according to treatment (Figure 1E–G); we indeed previously reported that the peak of CABG surgery‐induced inflammation was at POD4 (Noly et al. 2019).

First, baseline hs‐CRP levels measured 2 days before surgery were similar between placebo and quercetin‐treated patients (Table 1), suggesting that all patients presented the same high residual inflammatory risk. Second, a 2‐way repeated measures ANCOVA on the change of hs‐CRP levels revealed a significant time × treatment group interaction (*p* = 0.025). Treatment with quercetin during the perioperative period seems to have little effect on the inflammatory storm caused by the surgery in the ITT cohort, with a tendency to reduce hs‐CRP levels at hospital discharge (*p* = 0.073) but not earlier (POD1 *p* = 0.273; POD4 *p* = 0.422) (Figure 1B). Based on our recent data obtained in an independent cohort of CABG patients (Mury et al. 2024), we then explored sex differences in response to quercetin. Baseline hs‐CRP levels were similar between men and women, in both placebo and quercetin groups (Table S2). In the placebo group, women exhibited lower levels of hs‐CRP throughout the hospital stay post‐CABG, in particular at POD1 (women (W) = 87.3 ± 10.4 mg/L vs. men (M) = 112.8 ± 6.4 mg/L; *p* = 0.043) and POD4 (W = 84.97 ± 10.3 mg/L vs. M = 126.9 ± 7.8 mg/L; *p* = 0.018) (Figure 1C). In the quercetin group, however, hs‐CRP levels in women were comparable to those of men (Figure 1C), suggesting some pro‐inflammatory activity of quercetin in women, particularly at POD4 (quercetin = 120.4 ± 20.6 mg/L vs. placebo = 84.97 ± 10.3 mg/L; *p* = 0.149) (Figure 1D). In contrast, quercetin had overall limited effects on hs‐CRP in men, with only a tendency to reduce hs‐CRP levels at hospital discharge (quercetin = 45.9 ± 9.7 mg/L vs. placebo = 75.5 ± 18.0 mg/L, *p* = 0.083; Figure 1D).

Quercetin treatment had no statistically significant effect in the global population on circulating inflammatory factors determined by targeted proteomic at POD4 (Figure 1F). However, we identified numerous differentially expressed proteins (DEP) between sexes (Figure 1G). In the placebo population, men (M) exhibited a larger number of DEP than women (W) (M = 58 DEP vs. W = 6 DEP), suggesting a higher inflammatory proteomic signature in placebo men after 4 days of surgery, in accordance with higher hs‐CRP levels in placebo men (Figure 1C). In contrast, with quercetin treatment, men displayed a lower number of DEP than women (M = 10 DEP vs. W = 22 DEP) (Figure 1G), suggesting a pro‐inflammatory effect of quercetin in females, a tendency also seen with hs‐CRP levels at POD4 (*p* = 0.149, Figure 1D). In quercetin‐treated women, the proteomic analysis revealed the production of chemokines of T and B cells (i.e., CCL21, CCL28, CXCL9), inflammation (i.e., TNFSF13, INFG) and fibrosis (i.e., TGFA) (Figure 1G), all leading to a pro‐inflammatory deleterious effect when chronically increased. Compared to women, the inflammatory signature of men treated with quercetin was associated with markers suggestive of a better metabolism (i.e., SHMT1, METAP1D) and repair (i.e., PROK1, IL3RA) (Figure 1G); otherwise, quercetin had a less pronounced effect in men than in women, with only 10 DEP identified in men. Altogether, these data suggest that quercetin paradoxically increased multiple markers of inflammation in female patients, unlike in males where the effect of quercetin was more limited but beneficial.

### 3.4 Effect of a 2‐Day Quercetin Treatment on Endothelial Relaxant Function

**FIGURE 2:**
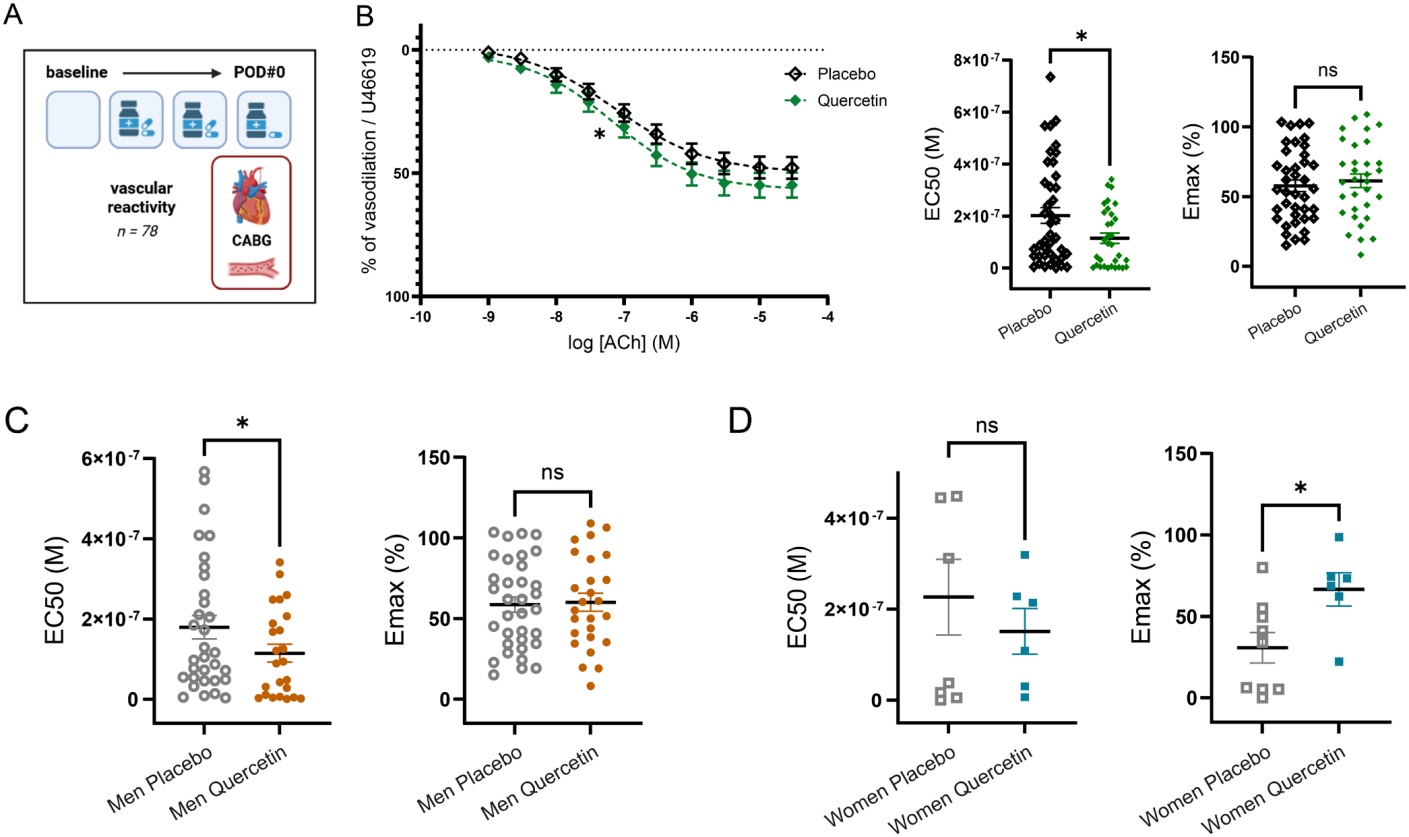
Impact of quercetin on endothelial relaxant function to acetylcholine. (A) Flow‐chart of the vascular endothelial function analysis performed the day of the surgery, after 2 days of exposure to quercetin (created using BioRender). (B) Endothelial‐dependent relaxation to ACh was assessed ex vivo on pre‐constricted arterial segments of the ITT population made available by the surgeons. EC_50_, half maximal effective concentration of ACh; E_max_, maximal effect of ACh. Values are expressed as mean ± SEM of *n* = 34 quercetin patients and *n* = 44 placebo patients arterial rings. A Mann–Whitney test was used to compare groups. *p < 0.05 vs. placebo. (C) Values are expressed as mean ± SEM of *n* = 28 men quercetin patients and *n* = 35 men placebo patients arterial rings. A Mann–Whitney test was used to compare groups. *p < 0.05 vs. men placebo. (D) Values are expressed as mean ± SEM of *n* = 6 women quercetin patients and *n* = 9 women placebo patients arterial rings. A Mann–Whitney test was used to compare groups. *p < 0.05 vs. women placebo.

Two days after the initiation of the treatment, we measured ex vivo endothelium‐dependent relaxation to acetylcholine (ACh) in an isolated segment of ITA, in 78 patients (Figure 2A). Briefly, isometric changes in tension were measured in pre‐constricted arterial segments and endothelium‐dependent relaxations were assessed by cumulative addition of increasing concentrations (from 1 nM to 10 μM) of ACh. Two typical indexes of endothelium‐dependent relaxation were measured: ACh‐EC_50_ to characterize the endothelial sensitivity to ACh (the lower the concentration of ACh needed to reach 50% of relaxation, the better the endothelial sensitivity), and E_max_ (the maximal relaxation of the ITA induced by ACh) to characterize the efficacy of ACh. ACh‐EC_50_ was significantly lower (better sensitivity) in the quercetin group (*n* = 44) than in the placebo (*n* = 34) group (median quercetin = 101 nM [6.48–228] vs. placebo = 151 nM [47.7–408.5]; *p* = 0.049), while E_max_ was similar between groups (Figure 2B). Because our previous observational study reported a higher endothelial dysfunction in male arteries (Mury et al. 2024), we explored whether sex was a defining variable of the response to quercetin: ACh‐EC_50_ was reduced by quercetin in male (Figure 2C, *p* = 0.043) but not in female arteries (Figure 2D, *p* = 0.852). In other words, quercetin improved endothelial sensitivity to ACh in men, but not in women. In women, however, quercetin increased ACh‐E_max_ (Figure 2D), while this parameter was not affected in men (Figure 2C). However, only the reduction in ACh‐EC_50_ reflects a physiological beneficial effect; E_max_ is indeed an extra‐physiological relaxant response only reached at high pharmacological doses of ACh. These results demonstrate that a 2‐day treatment with quercetin is sufficient to improve the endothelial sensitivity to ACh, but in male CAD patients only. Altogether, these data suggest a sex‐dependent effect of quercetin on endothelium‐dependent relaxant function consistent with a sex‐dependent reduction in systemic inflammation.

### 3.5 Identification of a 2‐Day Treatment Effect of Quercetin at the Transcriptomic Level

**FIGURE 3:**
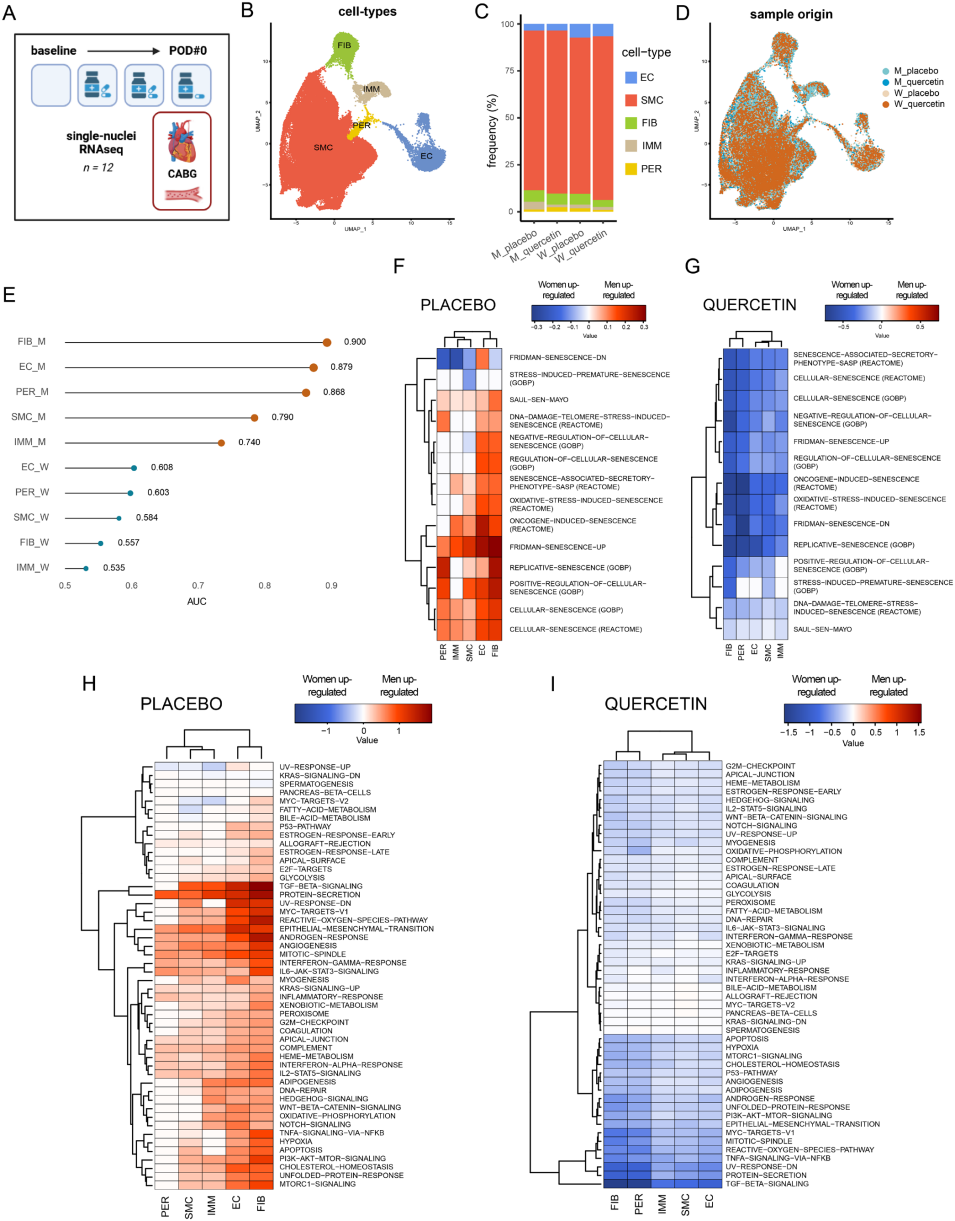
Sex‐dependent effect of quercetin treatment on single‐nuclei RNA sequencing. (A) Flow‐chart of the transcriptomic analysis using single‐nuclei RNA sequencing, performed on 3 samples per group collected the day of the surgery, after 2 days of quercetin treatment (created using BioRender). (B) Uniform Manifold Approximation and Projection (UMAP) plot, in 2 dimensions (UMAP‐1 and UMAP‐2), of annotated cell‐types clustered based on their specific gene expression, present in ITA graft arteries. (C) Proportion (%) of the 5 cell‐types (EC: Endothelial cells; SMC: Smooth muscle cells; FIB: Fibroblasts; IMM: Immune cells; PER: Pericytes) in men (M) and women (W) arteries, in placebo and quercetin groups. (D) UMAP plot colored by sample in men and women in placebo and quercetin groups. (E) Cell type prioritization representation of AUGUR predictive score, reflecting the effect of quercetin on the transcriptomic response in each arterial cell type, in men cells (brown circles) and in women cells (green circles); the higher the score (AUC), the higher the impact of quercetin. (F) Heatmap showing Gene Set Variation Analysis (GSVA) analysis of selected senescence‐related genes in the 5 cell types between men placebo and women placebo (F) and between men and women treated with quercetin (G). Rows represent gene‐related pathways (log2 fold change), and columns represent cell‐types. The color of each cell in the matrix represents the expression level of a specific pathway: Red corresponds to higher expression in men, and blue represents higher expression in women. On the heatmap, the dendrogram shows the hierarchical clustering of gene pathways based on the similarity of their expression profiles. H.eatmap showing GSVA analysis of Hallmark collection in the 5 cell types between men placebo and women placebo (H) and between men and women treated with quercetin (I).

To deepen our understanding of the effect of quercetin and the differential impact between sexes, we performed snRNA‐seq on ITA segments from a blind random selection of 12 patients *per* protocol of the cohort, including 3 men and 3 women from the placebo group, and 3 men and 3 women from the quercetin group (Figure 3A). In this reduced cohort, among the various clinical characteristics of the patients, only BMI showed a significant difference in the dataset we used (i.e., metadata; see supplemental Methods, ‘*Differential expression analysis and gene set variation analysis*’). BMI values were as follows: M placebo = 34.2 ± 1.6 kg/m^2^, M quercetin = 27.9 ± 1.4 kg/m^2^, W placebo = 38.1 ± 3.1 kg/m^2^, W querceti*n* = 28.0 ± 2.8 kg/m^2^ (*p* = 0.039), and this was accounted for in the analysis.

After quality control, we analyzed data from 79,201 single nuclei (M placebo = 28,504 vs. M quercetin = 16,988 vs. W placebo = 12,346 vs. W quercetin = 21,363 nuclei), with an average of 1089 genes expressed *per* nucleus. We clustered cells using unsupervised graph‐based clustering and visualized them as a uniform manifold approximation and projection (UMAP) graph to identify cell types (Figure 3B). Using canonical markers and automatic annotation (Mury et al. 2024), five large cell types uniformly distributed were identified between treatments and sexes: smooth muscle cells (SMC: 85.7%), fibroblasts (FIB: 5.4%), endothelial cells (EC: 4.9%), immune cells (IMM: 2.5%) and pericytes (PER: 1.4%) (Figures 3B–D and S2). The frequency of cell types was similar within the different groups (Figure 3C,D).

To initiate our analysis, we first used AUGUR, a method that allows to “*rank or prioritize cell types according to biological perturbations*” (Skinnider et al. 2021), to understand which cell types, in both sexes, are the most responsive to quercetin treatment (Figure 3E): the higher the AUC value (the closer to 1.0), the stronger is the effect of quercetin on a given cell type (Squair et al. 2021). This unbiased analysis shows that, in males, with very high predictive scores (AUC > 0.85 in FIB, EC and PER, and AUC > 0.74 in SMC and IMM) quercetin had a highly significant effect on all cell types. In contrast, in females, low predictive scores (AUC ≤ 0.6) show that female cells are less affected by quercetin (Figure 3E). This analysis highlights a sex‐dependent effects of quercetin, i.e., a stronger impact of quercetin in male cells when compared to female cells. This is in accordance with the beneficial effect of quercetin in male patients on systemic inflammatory profile (Figure 1) and with the higher endothelial sensitivity to ACh (Figure 2).

Next, we examined the sex differences in transcriptomic pathways in arteries from placebo‐ and quercetin‐treated patients: we focused on 14 selected senescence‐related pathways (Figure 3F,G) and on 50 pathways in the Hallmark collection (Figure 3H,I) at the transcriptomic level using gene set variation analysis (GSVA), as previously described (Mury et al. 2024). The heatmaps in placebo patients clearly show that senescence (Figure 3F) and inflammatory (Figure 3H) pathways were upregulated in placebo male cells (red signals) compared to placebo female cells (blue signals), in all cell types of the vascular wall, confirming our recent independent data (Mury et al. 2024). On the other hand, quercetin uniformly downregulated senescence (Figure 3G) and inflammatory (Figure 3I) pathways in male cells and upregulated these pathways in female cells (only blue signals). This transcriptomic sexual dimorphism justifies the integration of sex as a variable in all analyses.

**FIGURE 4:**
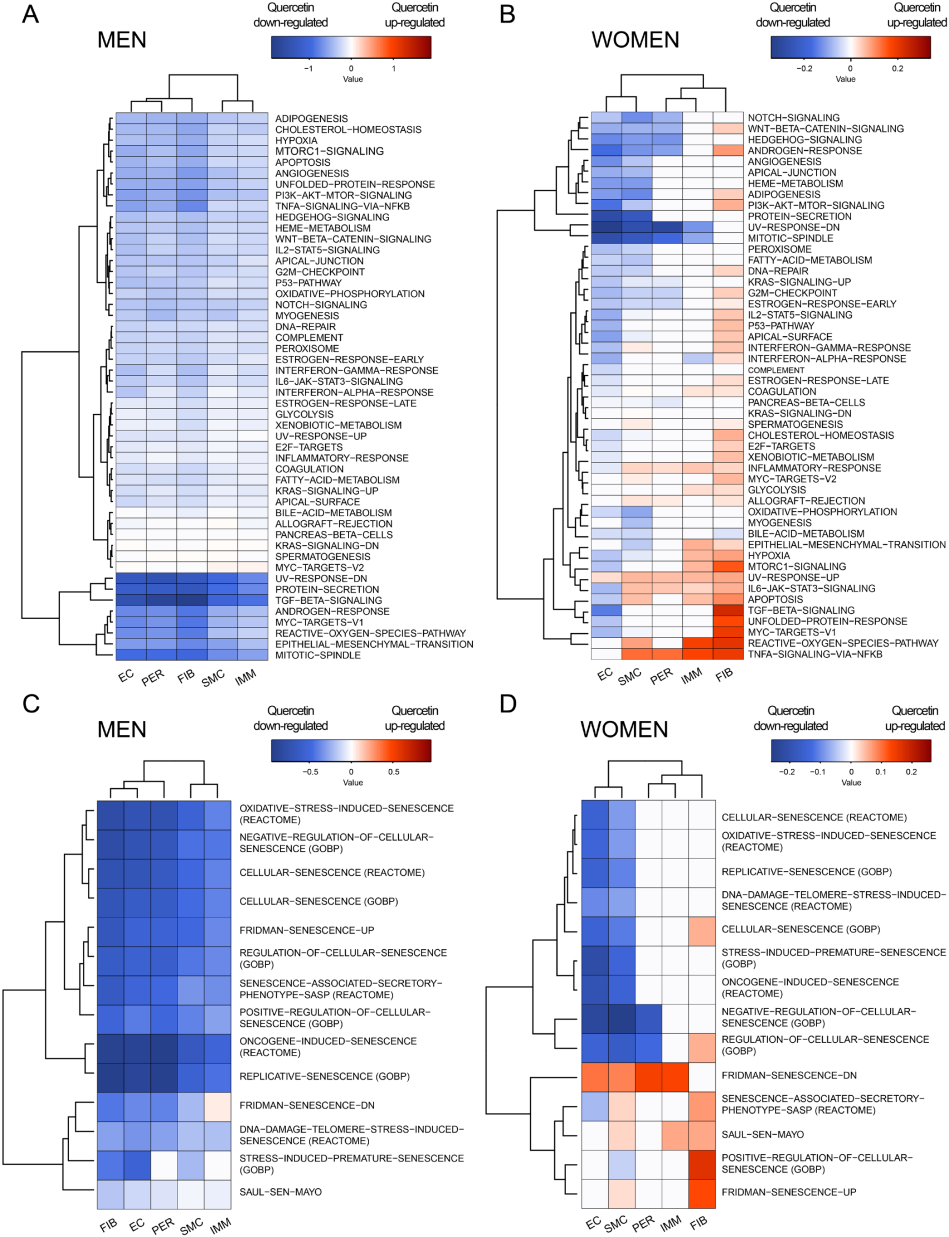
Pathway enrichment analysis reveals a uniform response to quercetin in male vascular cells and a cell type‐dependent response in female cells. (A) Heatmap showing GSVA analysis of Hallmark collection in the 5 cell types between men placebo and men quercetin. (B) Heatmap showing GSVA analysis of Hallmark collection in the 5 cell types between women placebo and women quercetin. (C) Heatmap showing GSVA analysis of selected senescence‐related gene sets in the 5 cell types between men placebo and men quercetin. (D) Heatmap showing GSVA analysis of selected senescence‐related gene sets in the 5 cell types between women placebo and women quercetin. Red and blue squares represent up‐regulated and down‐regulated pathways by quercetin, respectively.

We therefore specifically investigated the impact of sex on the response to quercetin (Figure 4). In male cells, for each arterial cell type, the heatmaps illustrating the effect of quercetin are almost all blue, representing down‐regulation of gene pathways by the senolytic (Figure 4A,C). This suggests that in male vascular cells from CAD patients, a 2‐day quercetin treatment displayed a strong anti‐inflammatory and anti‐senescence effect. In female cells, the GSVA results were more complex (Figure 4B,D). The response to quercetin was much lower in amplitude compared to men (Hallmark collection: changes from −0.2 to +0.2 in women, vs. changes from −1 to +1 in men, Figure 4A,B; Senescence: changes from −0.2 to +0.2 in women vs. changes from −0.5 to +0.5 in men, Figure 4C,D). Second, the response to quercetin in female cells was heterogenous and cell type‐dependent (Figure 4B,D): in EC and to a lesser extend in SMC, quercetin inhibited most of the Hallmark and senescence pathways (e.g., regulation of cellular process pathways like ‘*Notch signaling*’, ‘*Wnt beta catenin signaling*’, ‘*Hedgehog signaling*’, ‘*Cellular senescence*’, ‘*Oxidative stress induced senescence*’, among others). In contrast, in FIB from female quercetin patients, red signals are highlighted in ~70% of the 50 Hallmark pathways and in 43% of the senescence pathways, demonstrating that quercetin stimulated the *inflammaging* in this cell type. Indeed, inflammation‐related pathways like ‘*TNFα via NFκB signaling*’, ‘*Reactive oxygen species pathway*’, ‘*TGFβ signaling*’, ‘*Apoptosis*’, or ‘*IL6‐JAK‐STAT3 signaling*’ were more expressed in quercetin female cells than placebo female cells (Figure 4B). Similarly, in the senescence‐associated pathways, ‘*Positive regulation of cellular senescence*’, ‘*Fridman senescence up*’, ‘*Senescence associated secretory phenotype*’, and ‘*Cellular senescence*’ pathways were upregulated only in female FIB (Figure 4D). In PER and IMM female cells, the effect of quercetin was heterogeneous, with mostly no change (white signals), and some up‐ and down‐regulation of the Hallmark and senescence pathways (Figure 4B,D). Therefore, the global anti‐inflammatory and anti‐senescent effects of quercetin on the transcriptomic signature in male vascular cells are consistent with the reduced systemic inflammation and improved endothelial relaxant sensitivity in male arteries (Figures 1 and 2). The transcriptomic data also suggest that “*cooling*” of inflammatory and senescence pathways in all vascular cell types may be necessary to obtain a beneficial effect of quercetin on endothelial relaxant function. In addition, these results highlight both a sex‐ and cell‐specificity of quercetin, with a sex‐independent response in EC, but an opposite sex‐dependent response in FIB, as highlighted in Figure S3.

We further validated that quercetin reduced senescent load by promoting apoptosis of senescent cells, as expected from a senolytic drug. We focused on transcriptomic expression of two senescent markers, p21 (*CDKN1A*) and β‐galactosidase (*GLB1*) and on the apoptotic markers *BCL‐2*, *Bcl‐X/L1*, and *BAX*. Transcripts were measured in arterial wall cells, all cell types combined (Table 3), and in endothelial cells (Table 4), according to sex. In male arterial cells, quercetin significantly decreased both *CDKN1A* and *GLB1* transcripts and lowered expression of the anti‐apoptotic *Bcl‐XL1* (Table 3). Pro‐apoptotic *BAX* was expressed at a very low level and was also reduced by quercetin (Table 3). Altogether, these data support the concept that the senolytic quercetin eliminates male senescent arterial cells by inducing their apoptosis by lifting the anti‐apoptotic effect of Bcl proteins. In contrast, in female arterial cells, quercetin significantly increased *CDKN1A* while slightly reducing weakly expressed *GLB1* (Table 3). Anti‐apoptotic *BCL2L1* was significantly reduced, with no change in *BAX* expression (Table 3). Altogether, these analyses suggest that in female cells, quercetin increased *p21* expression in the arterial wall, suggestive of promotion of senescence, despite its pro‐apoptotic effect.

**TABLE 3:** Impact of quercetin on gene expression (snRNA‐seq) of senescence and apoptotic markers in combined arterial cells from male and female patients treated with placebo or quercetin.

Arterial cells	Quercetin	Placebo	% Q	% P	*p*	avglog_2_FC	*p* val adj
Males							
*CDKN1A*	0.150	0.228	0.100	0.162	1.54E‐74	−0.094	3.93E‐70
*GLB1*	0.060	0.083	0.039	0.073	2.11E‐47	−0.031	5.37E‐43
*BCL2*	0.231	0.520	0.107	0.297	0	−0.305	0
*BCL2L1*	0.052	0.087	0.031	0.076	3.98E‐85	−0.048	1.01E‐80
*BAX*	0.011	0.022	0.007	0.021	6.21E‐28	−0.016	1.58E‐23
Females							
*CDKN1A*	0.313	0.180	0.200	0.130	1.18E‐62	0.155	3.00E‐58
*GLB1*	0.069	0.086	0.056	0.073	3.06E‐09	−0.023	7.80E‐05
*BCL2*	0.421	0.368	0.217	0.209	0.013	0.054	1
*BCL2L1*	0.058	0.102	0.046	0.086	1.83E‐48	−0.059	4.67E‐44
*BAX*	0.017	0.018	0.014	0.016	0.141	−0.002	1

Abbreviations: % P, percentage of cells expressing the transcript in placebo; % Q, percentage of cells expressing the transcript in quercetin; avglog_2_FC, average log2 fold change in expression between quercetin and placebo condition; BAX, BCL2 associated X, apoptosis regulator; BCL2, BCL2 apoptosis regulator; BCL2L1, Bcl‐X; CDKN1A, cyclin dependent kinase inhibitor 1A (p21); GLB1, β‐galctosidase‐1; *p* val adj, *p* value adjusted after Bonferroni correction.

**TABLE 4:** Impact of quercetin on gene expression (snRNA‐seq) of senescence and apoptotic markers in endothelial cells (EC) from male and female patients treated with placebo or quercetin.

EC	Quercetin	Placebo	% Q	% P	*p* value	avglog_2_FC	*p* val adj
Males							
*CDKN1A*	0.104	0.545	0.062	0.312	4.04E‐31	−0.484	1.03E‐26
*GLB1*	0.061	0.093	0.042	0.084	0.001	−0.044	1
*BCL2*	0.064	0.073	0.029	0.058	0.010	−0.013	1
*BCL2L1*	0.067	0.156	0.039	0.138	5.03E‐10	−0.115	1.28E‐05
*BAX*	0.022	0.031	0.013	0.03	0.04	−0.012	1
Females							
*CDKN1A*	0.458	0.370	0.251	0.253	0.743	0.090	1
*GLB1*	0.063	0.092	0.051	0.074	0.022	−0.038	1
*BCL2*	0.066	0.015	0.037	0.014	0.001	0.070	1
*BCL2L1*	0.103	0.170	0.077	0.13	3.33E‐05	−0.086	0.848
*BAX*	0.025	0.018	0.018	0.015	0.663	0.010	1

Abbreviations: % P, percentage of cells expressing the transcript in placebo; % Q, percentage of cells expressing the transcript in quercetin; avglog_2_FC, average log2 fold change in expression between quercetin and placebo condition; BAX, BCL2 associated X, apoptosis regulator; BCL2, BCL2 apoptosis regulator; BCL2L1, Bcl‐X; CDKN1A, cyclin dependent kinase inhibitor 1A (p21); GLB1, β‐galctosidase‐1; *p* val adj, *p* value adjusted after Bonferroni correction.

When performed specifically in EC, the same analysis shows that *CDKN1A* and *BCL2L1* transcripts were also reduced significantly by quercetin in male EC, but no changes occurred in female EC (Table 4). Sex is therefore determinant in the senolytic effects of quercetin.

**FIGURE 5:**
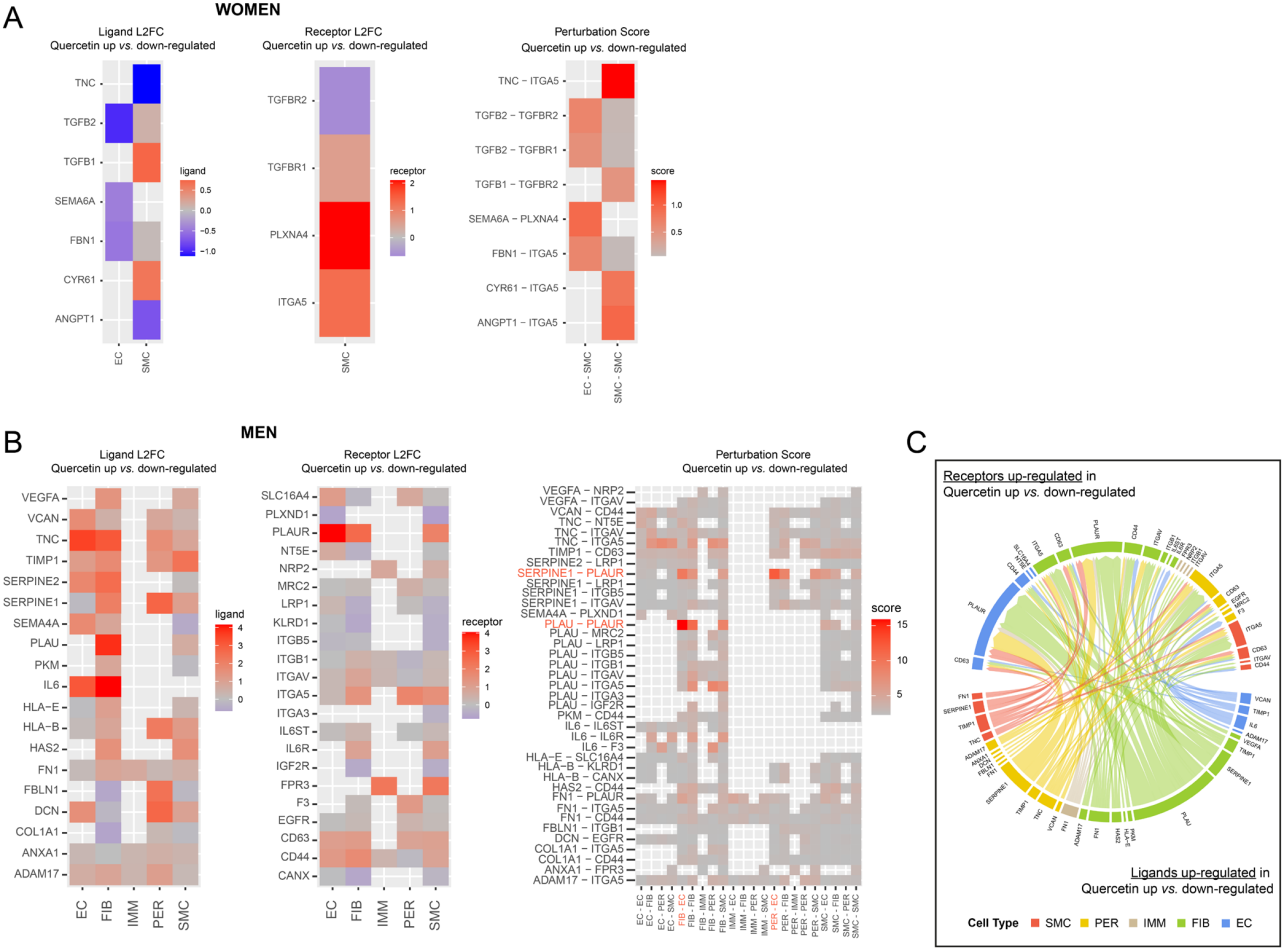
Identification of perturbed cell‐to‐cell interactions on the cell types the most affected by quercetin. (A) Differential expression and perturbation scores of ligands and receptors in women patients between placebo and quercetin groups. The left panel shows significant log2 fold changes (L2FC) of ligands in endothelial (EC) and smooth muscle cells (SMC), and the middle panel presents the significant L2FC for corresponding receptors in SMC. The right panel illustrates the perturbation scores, highlighting significant interactions among pathways between EC and SMC, and within SMC. (B) Differential expression and perturbation scores of ligands and receptors in men patients between placebo and quercetin groups. The left panel shows significant L2FC of ligands in all cell types of the wall, and the middle panel presents the L2FC for corresponding receptors. The right panel illustrates the perturbation scores, highlighting significant interactions among pathways in paracrine (cell‐to‐cell) and autocrine (one cell type) environment. (C) Circos Plot showing differential interactions illustrated in B (ligand up‐regulated by quercetin in the upper half, and receptors up‐regulated by quercetin in the bottom half) among the 5 male cell types, with connecting lines representing significant communication links in the signaling network between cell types.

Finally, since vascular cells do not function as isolated entities– in opposition to cancer cells, we investigated molecular perturbations by integrating cell‐to‐cell interaction signals in both sexes and in response to quercetin (Figure 5). Using the Connectome R package, we calculated in each sex and in each cell type separately the differential variation in expression of ligands and receptors. This led to the acquisition of a perturbation score for each putative ligand‐receptor interaction between quercetin and placebo groups. In female arteries, perturbation scores are low (score < 1.5), reflecting the low influence of quercetin (Figure 5A). In both EC and SMC from women, ligands highlighted in this analysis were mostly downregulated in the quercetin group (i.e., *TNC*, *TGFB2*, *SEMA6A*, *FBN1*, *ANGPT1*) (Figure 5A). Interestingly, 3 out of the 8 ligand‐receptor duos involve *TGFB* (isoform 1–2 as ligands, and isoform of receptor 1–2 as targets); a significant perturbation score in these duos, combined with downregulated *TGFB* ligands, suggests an inhibition of the TGF‐β signaling pathway in female EC, as initially observed in Figure 4B.

In contrast, in male EC and FIB, and to a lesser extent in PER, the analysis revealed more and stronger perturbed interactions (score > 15 in men vs. score > 1.5 in women) due to overexpression of ligands in the quercetin group (i.e., *TNC* in EC, *PLAU* and *IL6* in FIB and *SERPINE1* in PER) (Figure 5B). The highest perturbations therefore involved the 3 cell types identified by the AUGUR prediction, supporting their implication in the response to quercetin in men. Among the most differentially expressed interactions in these 3 cell types, *PLAUR*, encoding for the urokinase plasminogen activator receptor (uPAR), is the receptor with the highest scores of perturbations (Figure 5B) and the highest number of ligands (Figure 5C). The two main ligands of *PLAUR* are *PLAU* (encoding urokinase plasminogen activator, uPA) and *SERPINE1* (encoding plasminogen activator inhibitor, PAI‐1) in FIB and PER, respectively (Figure 5B,C). Thus, *PLAUR* could be one molecular target of quercetin to promote elimination of senescent cells in men.

## 4 Discussion

This double‐blind, randomized clinical trial is the first to identify quercetin as a potent vascular “*anti‐inflammaging*” agent in patients with symptomatic CAD. Quercetin was selected based on evidence demonstrating its short‐term (48‐h) senolytic activity on cultured senescent human endothelial cells (ECs) (Zhu et al. 2017, 2015) and its established safety profile (Dagher, Mury, Thorin‐Trescases, et al. 2021). Quercetin is a natural product, with reported anti‐inflammatory, anti‐oxidative, anti‐platelet, anti‐diabetic, anti‐bacterial, among other beneficial properties, and thus displays cardiovascular protective effects (Dagher, Mury, Thorin‐Trescases, et al. 2021). Our findings show that quercetin decreased post‐surgery incidence of new onset AF, significantly reduced post‐operative systemic inflammation, improved endothelial relaxant sensitivity to ACh, and reduced the canonical *inflammaging* transcriptomic signature—a composite of senescence and inflammatory pathways. These effects, however, were primarily observed in male patients. Thus, this study uniquely demonstrates a sex‐dependent clinical, functional, and molecular response to quercetin in CAD patients.

The most impactful clinical effect of the senolytic treatment was the reduced incidence of post‐operative AF in quercetin‐treated patients. Although this finding was unexpected considering the relatively low incidence (~15%) of AF in post‐CABG‐only patients, the small cohort in this study and the short‐term duration of the treatment with quercetin, these data further support the hypothesis that senescence drives AF (Mehdizadeh et al. 2022). Indeed, our recent preclinical studies and data from other groups (reviewed in (Mehdizadeh et al. 2022)), indicate that cellular senescence promotes AF susceptibility in animal models, and in cardiac patients, senescence in atrial tissues correlates with AF independently of old age (Mehdizadeh et al. 2024). Therefore, AF may be driven by cellular senescence and inflammation and is sensitive to the senolytic quercetin. We could not, however, determine the relationship between baseline parameters and AF occurrence because of the low rate of events. This finding must be validated in a larger cohort, since post‐surgery new onset AF is a known serious complication after a CABG procedure (Greenberg et al. 2017).

The primary endpoint of this clinical study was to test the anti‐inflammatory properties of quercetin in CAD patients. High sensitivity CRP tended to decrease at hospital discharge only, suggesting that quercetin is a weak anti‐inflammatory, not able to counteract the surge of hs‐CRP induced by the surgery. Patients had acute ACS within the last 30 days, indicated by high baseline levels of hs‐CRP (> 7 mg/L). However, unexpected results were observed in female patients: first, in the placebo groups, women showed smaller increases in post‐operative hs‐CRP than men, while quercetin raised post‐operative hs‐CRP in women to levels measured in men. In the literature, baseline hs‐CRP is typically higher in women with CVD, possibly due to differences in autoimmune disease sensitivity, fat distribution, and estrogens levels, which can increase hs‐CRP (reviewed in Lobo and Jaffe 2023). In our study, women were post‐menopausal, with a similar BMI (around 30 kg/m^2^) to men. Similar high basal hs‐CRP levels may be explained by the fact that all patients had ACS in the past 30 days. The proteomic analysis provided more qualitative information. Compared to men, women expressed fewer inflammatory factors in the placebo groups, but this profile was reversed by quercetin. Therefore, the proteomic panel reflects the changes in hs‐CRP. The in‐depth analysis of the inflammatory response revealed that quercetin increased the potent pro‐inflammatory factors CXCL9 and INF‐γ in women. INF‐γ is the only known stimulus of CXCL9 (Giamarellos‐Bourboulis et al. 2024), a chemokine that has been reported to rise in women with autoimmune diseases who develop CAD (Shamsi et al. 2023). Therefore, the rise in CXCL9 and INF‐γ by quercetin in women is likely sex‐specific. In addition, while INF‐γ expression was elevated in women in both placebo and quercetin groups compared to men, the potent anti‐inflammatory cytokine IL‐10 involved in the resolution of inflammation (Saraiva et al. 2020) dropped in women treated with quercetin. In summary, short‐term treatment with quercetin unexpectedly increased inflammatory markers in women with CAD and had a beneficial, but limited, anti‐inflammatory effect in men based on our knowledge of the canonical *inflammaging* pathways (Mury et al. 2024). It remains to be tested whether the pro‐inflammatory response observed in women treated with quercetin reflects a transitory sex‐specific repair response.

Gene Set Variation Analysis reveals that in females FIB, quercetin weakly but significantly stimulated 70% of the Hallmark pathways and 43% of the senescence pathways. This contrasts with the anti‐inflammatory and anti‐senescent effect of quercetin observed in female EC and in all male cell types. The reason for this cell‐ and sex‐dependent effect of quercetin is unclear. Notably, deleterious effects of senolytics have been reported in female mice (Budamagunta et al. 2023; Fang et al. 2023; Schwab et al. 2022) and rats (Rani et al. 2024). Few transcriptomic or proteomic signatures of senolytics exist. For example, in mice with repeated traumatic brain injury, ABT‐263 (navitoclax) lowered p21 and p16 protein levels in males but increased p16 in females without affecting p21 (Schwab et al. 2022). We also found that navitoclax increased senescence and SASP markers *p21*, *Il6*, and *Pai‐1* expression in females but not males, improving cognition in males but worsening it in females with severe atherosclerosis (Lambert et al. 2025). These data suggest that senolytics have sex‐dependent effects, beneficial in males and potentially harmful in females.

Accordingly, quercetin also failed to improve endothelial sensitivity to ACh in female arteries, but significantly increased endothelial function in men, as expected from a senolytic with anti‐inflammatory properties (Dagher, Mury, Thorin‐Trescases, et al. 2021). Women in their mid‐fifties develop vascular endothelial dysfunction, but 10 years later than men (Celermajer et al. 1994; Lakatta et al. 2009; McEniery et al. 2006), and endothelial function remains better in older women than in men (Mury et al. 2024). Endothelial dysfunction and its associated *inflammaging* (Mury et al. 2024) being prodromal to CAD (Halcox et al. 2002; Shechter et al. 2009), the use of senolytics in women may need to be delayed to optimize their efficacy. A potential explanation for the deleterious (inflammation) or the absence of effect (endothelial function) of quercetin in women patients may reside at the molecular level (Mury et al. 2024).

In the transcriptomic analysis, AUGUR predictions showed that all male arterial cell types exhibited a strong response to quercetin (AUC ~ 0.9; perturbation scores > 15). Indeed, in male CAD patients, quercetin exposure induced a robust anti‐inflammatory and anti‐senescence transcriptomic response across all vascular cell types, particularly in ECs, FIBs, and PERs. In contrast, in female cells, the efficacy was lower (AUC ~ 0.6) with smaller perturbation scores (~1.5); although there was a small beneficial molecular effect in female ECs, quercetin increased inflammatory and senescence‐associated pathways in FIBs, with heterogeneous effects in other cell types. The origin of the heterogeneous and limited response to quercetin in female samples may come from the important differences in baseline transcriptomic profile between the two sexes. Indeed, the transcriptomic *inflammaging* signature in placebo arteries differed according to the sex of the patient, confirming our previous study in an independent cohort of CAD patients (Mury et al. 2024): compared to placebo female cells, placebo male vascular cells of all types exhibited higher and stronger inflammatory and senescent transcriptomic signatures. This strongly suggests that female vascular cells are “healthier” and may explain why female patients did not gain from quercetin, and why only male patients benefited from it. In other words, the senolytic effects of quercetin likely require a high level of cellular inflammation and senescence, primarily observed in male vascular cells.

In addition to being healthier, female cells like FIB, that responded to quercetin by a higher *inflammaging* signature, may use senescence and inflammatory pathways as active defense and repair mechanisms, explaining why inhibition of such pathways appears deleterious. In women, cellular senescence may function as a transient healing mechanism, while in men, chronic accumulation of senescent cells may reflect an unsuccessful attempt to repair/healing, rendering the senolytic effects of quercetin more effective. The literature is indeed clear on the fact that chronic accumulation of senescent cells with aging is deleterious likely through the SASP (Bloom et al. 2023; Liberale et al. 2022; Malaquin et al. 2016; Xu et al. 2018). Therefore, we propose that healthier female vascular cells from CAD women patients effectively use senescence as a repair/defense mechanism, in contrast to male cells.

The molecular target of quercetin in male EC, FIB, and PER includes interactions between the receptor *PLAUR* and its ligands *PLAU* and *SERPINE1*. The urokinase pathway is known to be activated in CAD patients and associated with endothelial dysfunction (Corban et al. 2018; Hamada et al. 2024). The Connectome analysis showed that quercetin *increased* the *PLAU/SERPINE1 → PLAUR* axis in male FIB‐EC and in male PER‐EC. Targeting PLAUR successfully favored immune senolysis in various animal models of senescent cell overload (Amor et al. 2020). This finding confirms a dual role for the urokinase pathway, potentially acting both as a repair and a senescent cell death mechanism. Such functional duality has been proposed in the context of senescence, where increased repair protein activity reflects the severity of the damage (Ogrodnik et al. 2019). Thus, quercetin‐dependent overexpression of the *PLAU/SERPINE1 → PLAUR* axis may yield complex outcomes, promoting both repair and cell death, leading ultimately to lower inflammation and senescence, associated with better endothelial function. The demonstration that putative interactions between PLAU/SERPINE1 and their receptor PLAUR are increased in men only will need to be validated in atherosclerotic mice of both sexes (Amor et al. 2020). In women, the TGFβ signaling pathway appears down‐regulated by quercetin; this pathway and the associated fibrous phenotype of the atherosclerotic plaque are known to be uniquely activated in female patients (Jin et al. 2021; Sakkers et al. 2023; Theofilatos et al. 2023). Altogether, our study suggests that the reduction in vascular senescent cell load in CAD patients by short‐term exposure to quercetin is more beneficial in men than in women.

## 5 Study Limitations

(1) With a 9‐day duration, this clinical trial is not a long‐term outcome study; this was not the aim of this study. Whether the changes in transcriptomic induced by short‐term exposure to quercetin will slow atherogenesis, limit future cardiovascular events, including AF, and draw a transcriptomic signature specific to AF, will need to be tested in a larger cohort of patients with a longer follow‐up. Yet, most events occur immediately post‐surgery and during the hospital stay and within the first month post‐CABG surgery (Heer et al. 2022). (2) In addition, the pro‐inflammatory and pro‐senescent effects of quercetin in women do not necessarily mean that quercetin is harmful in women, since, for example, none developed new post‐operative AF. Transitory inflammation is a vital repair mechanism (Straub and Schradin 2016). (3) The low number of female patients recruited is a limitation, representing only ~20% of all patients; this reflects, however, the lower incidence of corrective CABG surgeries for CAD in women, a clinical fact with no clear explanation considering that acute myocardial infarction is the first cause of death in both men and women living in high‐income countries (Woodward 2019). (4) In the current study, statistical power analysis did not account for sex as a variable, which limited the statistical power of the data, particularly for the endothelial function parameters collected from female patients. Nonetheless, dissecting the data *per* sex from 4 different methodologies consistently highlights that women responded to quercetin differently than men. A larger, multicentric clinical trial including sex as a variable should be performed to validate our results. (5) In addition, because the arterial transcriptomic data do not simply reflect the systemic proteomic inflammation, it is very difficult to capture one global arterial and systemic inflammatory profile. (6) Another limitation of our study is the use of ITA segments that are not prone to atherosclerosis. Sex is a variable in the molecular determinant of plaque structure (Diez Benavente et al. 2023; Hartman et al. 2021; Sakkers et al. 2023) but there is no information on the pre‐plaque transcriptome of the endothelium of female and male coronary arteries. It is important to remind that these patients had a recent ACS and that they may differ from patients with stable angina in terms of baseline inflammatory signature.

## 6 Conclusion

Quercetin exposure in symptomatic CAD patients decreased AF incidence, reduced post‐operative inflammation, improved endothelial relaxant sensitivity to ACh, and mitigated the canonical *inflammaging* transcriptomic signature. However, these benefits were predominantly observed in male patients. This trial demonstrates the potential of quercetin as a vascular “*anti‐inflammaging*” agent while highlighting the need for further investigation into sex‐specific responses. Additional longitudinal clinical studies are now needed to test whether intermittent exposure to quercetin over longer follow‐up periods would reduce the number of major adverse cardiovascular events in patients with CVD, and whether sex would be a determinant variable.

## Author Contributions

Conceptualization, experimentation, methodology, patient recruitment, data analysis, manuscript writing, manuscript revision: P.M. Clinical design, patient recruitment, manuscript revision: O.D. Clinical data statistical analysis, manuscript revision: A.F. Clinical design, patient recruitment, manuscript revision: A.D., Y.L., P.‐E.N., M.I., P.P., P.D., D.B., P.‐L.B., N.P., E.M., N.D., H.J., M.P. Single nuclei RNA‐seq supervision, data revision, data analysis, manuscript revision: G.L. Data analysis, manuscript writing, manuscript revision: N.T.‐T. Clinical design: M.C. Conceptualization, experimentation, methodology, patient recruitment, data monitoring, manuscript writing, manuscript revision: E.T.

## Conflicts of Interest

The authors declare no conflicts of interest.

## Supporting information


**Data S1.** Supporting Information.

## Data Availability

All single‐nucleus RNA sequencing data for the study have been deposited in the NCBI's Gene Expression Omnibus database (GSE278420).

## References

[acel70108-bib-0001] Amor, C. , J. Feucht , J. Leibold , et al. 2020. “Senolytic CAR T Cells Reverse Senescence‐Associated Pathologies.” Nature 583, no. 7814: 127–132. 10.1038/s41586-020-2403-9. 32555459 PMC7583560

[acel70108-bib-0002] Bloom, S. I. , M. T. Islam , L. A. Lesniewski , and A. J. Donato . 2023. “Mechanisms and Consequences of Endothelial Cell Senescence.” Nature Reviews. Cardiology 20, no. 1: 38–51. 10.1038/s41569-022-00739-0. 35853997 PMC10026597

[acel70108-bib-0003] Budamagunta, V. , A. Kumar , A. Rani , et al. 2023. “Effect of Peripheral Cellular Senescence on Brain Aging and Cognitive Decline.” Aging Cell 22, no. 5: e13817. 10.1111/acel.13817. 36959691 PMC10186609

[acel70108-bib-0004] Caldemeyer, L. , M. Dugan , J. Edwards , and L. Akard . 2016. “Long‐Term Side Effects of Tyrosine Kinase Inhibitors in Chronic Myeloid Leukemia.” Current Hematologic Malignancy Reports 11, no. 2: 71–79. 10.1007/s11899-016-0309-2. 26922746

[acel70108-bib-0005] Celermajer, D. S. , K. E. Sorensen , D. J. Spiegelhalter , D. Georgakopoulos , J. Robinson , and J. E. Deanfield . 1994. “Aging Is Associated With Endothelial Dysfunction in Healthy Men Years Before the Age‐Related Decline in Women.” Journal of the American College of Cardiology 24, no. 2: 471–476. 10.1016/0735-1097(94)90305-0. 8034885

[acel70108-bib-0006] Childs, B. G. , D. J. Baker , T. Wijshake , C. A. Conover , J. Campisi , and J. M. van Deursen . 2016. “Senescent Intimal Foam Cells Are Deleterious at All Stages of Atherosclerosis.” Science 354, no. 6311: 472–477. 10.1126/science.aaf6659. 27789842 PMC5112585

[acel70108-bib-0007] Childs, B. G. , M. Gluscevic , D. J. Baker , et al. 2017. “Senescent Cells: An Emerging Target for Diseases of Ageing.” Nature Reviews. Drug Discovery 16, no. 10: 718–735. 10.1038/nrd.2017.116. 28729727 PMC5942225

[acel70108-bib-0008] Coppé, J. P. , P. Y. Desprez , A. Krtolica , and J. Campisi . 2010. “The Senescence‐Associated Secretory Phenotype: The Dark Side of Tumor Suppression.” Annual Review of Pathology 5: 99–118. 10.1146/annurev-pathol-121808-102144. PMC4166495 20078217

[acel70108-bib-0009] Corban, M. T. , A. Prasad , L. Nesbitt , et al. 2018. “Local Production of Soluble Urokinase Plasminogen Activator Receptor and Plasminogen Activator Inhibitor‐1 in the Coronary Circulation Is Associated With Coronary Endothelial Dysfunction in Humans.” Journal of the American Heart Association 7, no. 15: e009881. 10.1161/JAHA.118.009881. 30371230 PMC6201458

[acel70108-bib-0010] Dagher, O. , P. Mury , P. E. Noly , et al. 2021. “Design of a Randomized Placebo‐Controlled Trial to Evaluate the Anti‐Inflammatory and Senolytic Effects of Quercetin in Patients Undergoing Coronary Artery Bypass Graft Surgery.” Frontiers in Cardiovascular Medicine 8: 741542. 10.3389/fcvm.2021.741542. 34746258 PMC8564044

[acel70108-bib-0011] Dagher, O. , P. Mury , N. Thorin‐Trescases , P. E. Noly , E. Thorin , and M. Carrier . 2021. “Therapeutic Potential of Quercetin to Alleviate Endothelial Dysfunction in Age‐Related Cardiovascular Diseases.” Frontiers in Cardiovascular Medicine 8: 658400. 10.3389/fcvm.2021.658400. 33860002 PMC8042157

[acel70108-bib-0012] Diez Benavente, E. , S. Karnewar , M. Buono , et al. 2023. “Female Gene Networks Are Expressed in Myofibroblast‐Like Smooth Muscle Cells in Vulnerable Atherosclerotic Plaques.” Arteriosclerosis, Thrombosis, and Vascular Biology 43, no. 10: 1836–1850. 10.1161/ATVBAHA.123.319325. 37589136 PMC10521798

[acel70108-bib-0013] Fang, Y. , D. Medina , R. Stockwell , et al. 2023. “Sexual Dimorphic Metabolic and Cognitive Responses of C57BL/6 Mice to Fisetin or Dasatinib and Quercetin Cocktail Oral Treatment.” Geroscience 45, no. 5: 2835–2850. 10.1007/s11357-023-00843-0. 37296266 PMC10643448

[acel70108-bib-0014] Franceschi, C. , M. Bonafe , S. Valensin , et al. 2000. “Inflammaging. An Evolutionary Perspective on Immunosenescence.” Annals of the New York Academy of Sciences 908: 244–254. 10.1111/j.1749-6632.2000.tb06651.x. 10911963

[acel70108-bib-0015] Giamarellos‐Bourboulis, E. J. , M. Antonelli , F. Bloos , et al. 2024. “Interferon‐Gamma Driven Elevation of CXCL9: A New Sepsis Endotype Independently Associated With Mortality.” eBioMedicine 109: 105–414. 10.1016/j.ebiom.2024.105414. PMC11539129 39447386

[acel70108-bib-0016] Gonzales, M. M. , V. R. Garbarino , T. F. Kautz , et al. 2023. “Senolytic Therapy in Mild Alzheimer's Disease: A Phase 1 Feasibility Trial.” Nature Medicine 29, no. 10: 2481–2488. 10.1038/s41591-023-02543-w. PMC10875739 37679434

[acel70108-bib-0017] Greenberg, J. W. , T. S. Lancaster , R. B. Schuessler , and S. J. Melby . 2017. “Postoperative Atrial Fibrillation Following Cardiac Surgery: A Persistent Complication.” European Journal of Cardio‐Thoracic Surgery 52, no. 4: 665–672. 10.1093/ejcts/ezx039. 28369234

[acel70108-bib-0018] Halcox, J. P. , W. H. Schenke , G. Zalos , et al. 2002. “Prognostic Value of Coronary Vascular Endothelial Dysfunction.” Circulation 106, no. 6: 653–658. 10.1161/01.cir.0000025404.78001.d8. 12163423

[acel70108-bib-0019] Hamada, M. , K. S. Varkoly , O. Riyadh , et al. 2024. “Urokinase‐Type Plasminogen Activator Receptor (uPAR) in Inflammation and Disease: A Unique Inflammatory Pathway Activator.” Biomedicine 12, no. 6: 1167. 10.3390/biomedicines12061167. PMC11201033 38927374

[acel70108-bib-0020] Hartman, R. J. G. , K. Owsiany , L. Ma , et al. 2021. “Sex‐Stratified Gene Regulatory Networks Reveal Female Key Driver Genes of Atherosclerosis Involved in Smooth Muscle Cell Phenotype Switching.” Circulation 143, no. 7: 713–726. 10.1161/CIRCULATIONAHA.120.051231. 33499648 PMC7930467

[acel70108-bib-0021] Heer, T. , M. von Scheidt , A. Boening , et al. 2022. “Prognostic Impact of Secondary Prevention After Coronary Artery Bypass Grafting‐Insights From the TiCAB Trial.” European Journal of Cardio‐Thoracic Surgery 62, no. 3: ezac048. 10.1093/ejcts/ezac048. 35138350

[acel70108-bib-0022] Hickson, L. J. , L. G. P. Langhi Prata , S. A. Bobart , et al. 2019. “Senolytics Decrease Senescent Cells in Humans: Preliminary Report From a Clinical Trial of Dasatinib Plus Quercetin in Individuals With Diabetic Kidney Disease.” eBioMedicine 47: 446–456. 10.1016/j.ebiom.2019.08.069. 31542391 PMC6796530

[acel70108-bib-0023] Jin, H. , B. M. E. Mees , E. A. L. Biessen , and J. C. Sluimer . 2021. “Transcriptional Sex Dimorphism in Human Atherosclerosis Relates to Plaque Type.” Circulation Research 129, no. 12: 1175–1177. 10.1161/CIRCRESAHA.121.320099. 34661425

[acel70108-bib-0024] Kirkland, J. L. , T. Tchkonia , Y. Zhu , L. J. Niedernhofer , and P. D. Robbins . 2017. “The Clinical Potential of Senolytic Drugs.” Journal of the American Geriatrics Society 65, no. 10: 2297–2301. 10.1111/jgs.14969. 28869295 PMC5641223

[acel70108-bib-0025] Kuilman, T. , C. Michaloglou , W. J. Mooi , and D. S. Peeper . 2010. “The Essence of Senescence.” Genes & Development 24, no. 22: 2463–2479. 10.1101/gad.1971610. 21078816 PMC2975923

[acel70108-bib-0026] Lakatta, E. G. , M. Wang , and S. S. Najjar . 2009. “Arterial Aging and Subclinical Arterial Disease Are Fundamentally Intertwined at Macroscopic and Molecular Levels.” Medical Clinics of North America 93, no. 3: 583–604. 10.1016/j.mcna.2009.02.008. 19427493 PMC2943242

[acel70108-bib-0027] Lambert, M. , G. Miquel , L. Villeneuve , N. Thorin‐Trescases , and E. Thorin . 2025. “The Senolytic ABT‐263 Improves Cognitive Functions in Middle‐Aged Male, but Not Female, Atherosclerotic LDLr(−/−);hApoB(100)(+/+) Mice.” Geroscience. 10.1007/s11357-025-01563-3. Online ahead of print. PMC12181456 39982668

[acel70108-bib-0028] Liberale, L. , L. Badimon , F. Montecucco , T. F. Luscher , P. Libby , and G. G. Camici . 2022. “Inflammation, Aging, and Cardiovascular Disease: JACC Review Topic of the Week.” Journal of the American College of Cardiology 79, no. 8: 837–847. 10.1016/j.jacc.2021.12.017. 35210039 PMC8881676

[acel70108-bib-0056] Lobo, R. , and A. S. Jaffe . 2023. “Sex‐Specific Thresholds for Cardiac Biomarkers‐We Need to Move Forward.” Reviews in Cardiovascular Medicine 24, no. 3: 86. 10.31083/j.rcm2403086. 39077486 PMC11264030

[acel70108-bib-0029] Malaquin, N. , A. Martinez , and F. Rodier . 2016. “Keeping the Senescence Secretome Under Control: Molecular Reins on the Senescence‐Associated Secretory Phenotype.” Experimental Gerontology 82: 39–49. 10.1016/j.exger.2016.05.010. 27235851

[acel70108-bib-0030] McEniery, C. M. , S. Wallace , I. S. Mackenzie , et al. 2006. “Endothelial Function Is Associated With Pulse Pressure, Pulse Wave Velocity, and Augmentation Index in Healthy Humans.” Hypertension 48, no. 4: 602–608. 10.1161/01.HYP.0000239206.64270.5f. 16940223

[acel70108-bib-0031] McHugh, D. , and J. Gil . 2018. “Senescence and Aging: Causes, Consequences, and Therapeutic Avenues.” Journal of Cell Biology 217, no. 1: 65–77. 10.1083/jcb.201708092. 29114066 PMC5748990

[acel70108-bib-0032] Mehdizadeh, M. , M. Aguilar , E. Thorin , G. Ferbeyre , and S. Nattel . 2022. “The Role of Cellular Senescence in Cardiac Disease: Basic Biology and Clinical Relevance.” Nature Reviews. Cardiology 19, no. 4: 250–264. 10.1038/s41569-021-00624-2. 34667279

[acel70108-bib-0033] Mehdizadeh, M. , P. Naud , I. H. Abu‐Taha , et al. 2024. “The Role of Cellular Senescence in Profibrillatory Atrial Remodelling Associated With Cardiac Pathology.” Cardiovascular Research 120, no. 5: 506–518. 10.1093/cvr/cvae003. 38181429 PMC11060482

[acel70108-bib-0034] Mury, P. , G. Cagnone , O. Dagher , et al. 2024. “Senescence and Inflamm‐Aging Are Associated With Endothelial Dysfunction in Men but Not Women With Atherosclerosis.” JACC: Basic to Translational Science 9, no. 10: 1163–1177. 10.1016/j.jacbts.2024.06.012. 39534645 PMC11551873

[acel70108-bib-0035] Noly, P. E. , P. Labbe , N. Thorin‐Trescases , et al. 2019. “Reduction of Plasma Angiopoietin‐Like 2 After Cardiac Surgery Is Related to Tissue Inflammation and Senescence Status of Patients.” Journal of Thoracic and Cardiovascular Surgery 158, no. 3: 792–802. 10.1016/j.jtcvs.2018.12.047. 30745045

[acel70108-bib-0036] Ogrodnik, M. , H. Salmonowicz , and V. N. Gladyshev . 2019. “Integrating Cellular Senescence With the Concept of Damage Accumulation in Aging: Relevance for Clearance of Senescent Cells.” Aging Cell 18, no. 1: e12841. 10.1111/acel.12841. 30346102 PMC6351832

[acel70108-bib-0037] Rani, A. , L. Bean , V. Budamagunta , A. Kumar , and T. C. Foster . 2024. “Failure of Senolytic Treatment to Prevent Cognitive Decline in a Female Rodent Model of Aging.” Frontiers in Aging Neuroscience 16, no. 1: 384–554. 10.3389/fnagi.2024.1384554. PMC11133672 38813533

[acel70108-bib-0038] Ridker, P. M. , B. M. Everett , T. Thuren , et al. 2017. “Antiinflammatory Therapy With Canakinumab for Atherosclerotic Disease.” New England Journal of Medicine 377, no. 12: 1119–1131. 10.1056/NEJMoa1707914. 28845751

[acel70108-bib-0039] Ridker, P. M. , J. G. MacFadyen , B. M. Everett , P. Libby , T. Thuren , and R. J. Glynn . 2018. “Relationship of C‐Reactive Protein Reduction to Cardiovascular Event Reduction Following Treatment With Canakinumab: A Secondary Analysis From the CANTOS Randomised Controlled Trial.” Lancet 391, no. 10118: 319–328. 10.1016/s0140-6736(17)32814-3. 29146124

[acel70108-bib-0040] Ritchie, M. E. , B. Phipson , D. Wu , et al. 2015. “Limma Powers Differential Expression Analyses for RNA‐Sequencing and Microarray Studies.” Nucleic Acids Research 43, no. 7: e47. 10.1093/nar/gkv007. 25605792 PMC4402510

[acel70108-bib-0041] Roos, C. M. , B. Zhang , A. K. Palmer , et al. 2016. “Chronic Senolytic Treatment Alleviates Established Vasomotor Dysfunction in Aged or Atherosclerotic Mice.” Aging Cell 15, no. 5: 973–977. 10.1111/acel.12458. 26864908 PMC5013022

[acel70108-bib-0042] Sakkers, T. R. , M. Mokry , M. Civelek , et al. 2023. “Sex Differences in the Genetic and Molecular Mechanisms of Coronary Artery Disease.” Atherosclerosis 384: 117279. 10.1016/j.atherosclerosis.2023.117279. 37805337

[acel70108-bib-0043] Saraiva, M. , P. Vieira , and A. O'Garra . 2020. “Biology and Therapeutic Potential of Interleukin‐10.” Journal of Experimental Medicine 217, no. 1: e20190418. 10.1084/jem.20190418. 31611251 PMC7037253

[acel70108-bib-0044] Schwab, N. , D. Taskina , E. Leung , B. T. Innes , G. D. Bader , and L. N. Hazrati . 2022. “Neurons and Glial Cells Acquire a Senescent Signature After Repeated Mild Traumatic Brain Injury in a Sex‐Dependent Manner.” Frontiers in Neuroscience 16: 1027116. 10.3389/fnins.2022.1027116. 36408415 PMC9669743

[acel70108-bib-0045] Shamsi, A. , S. A. Roghani , Z. Abdan , et al. 2023. “CXCL9 and Its Receptor CXCR3, an Important Link Between Inflammation and Cardiovascular Risks in RA Patients.” Inflammation 46, no. 6: 2374–2385. 10.1007/s10753-023-01884-5. 37542661

[acel70108-bib-0046] Shechter, M. , A. Issachar , I. Marai , et al. 2009. “Long‐Term Association of Brachial Artery Flow‐Mediated Vasodilation and Cardiovascular Events in Middle‐Aged Subjects With no Apparent Heart Disease.” International Journal of Cardiology 134, no. 1: 52–58. 10.1016/j.ijcard.2008.01.021. 18479768

[acel70108-bib-0047] Skinnider, M. A. , J. W. Squair , C. Kathe , et al. 2021. “Cell Type Prioritization In Single‐Cell Data.” Nature Biotechnology 39, no. 1: 30–34. 10.1038/s41587-020-0605-1. PMC7610525 32690972

[acel70108-bib-0048] Squair, J. W. , M. A. Skinnider , M. Gautier , L. J. Foster , and G. Courtine . 2021. “Prioritization of Cell Types Responsive to Biological Perturbations in Single‐Cell Data With Augur.” Nature Protocols 16, no. 8: 3836–3873. 10.1038/s41596-021-00561-x. 34172974

[acel70108-bib-0049] Straub, R. H. , and C. Schradin . 2016. “Chronic Inflammatory Systemic Diseases: An Evolutionary Trade‐Off Between Acutely Beneficial but Chronically Harmful Programs.” Evolution, Medicine, and Public Health 2016, no. 1: 37–51. 10.1093/emph/eow001. 26817483 PMC4753361

[acel70108-bib-0050] Theofilatos, K. , S. Stojkovic , M. Hasman , et al. 2023. “Proteomic Atlas of Atherosclerosis: The Contribution of Proteoglycans to Sex Differences, Plaque Phenotypes, and Outcomes.” Circulation Research 133, no. 7: 542–558. 10.1161/CIRCRESAHA.123.322590. 37646165 PMC10498884

[acel70108-bib-0051] Wilson, W. H. , O. A. O'Connor , M. S. Czuczman , et al. 2010. “Navitoclax, a Targeted High‐Affinity Inhibitor of BCL‐2, in Lymphoid Malignancies: A Phase 1 Dose‐Escalation Study of Safety, Pharmacokinetics, Pharmacodynamics, and Antitumour Activity.” Lancet Oncology 11, no. 12: 1149–1159. 10.1016/S1470-2045(10)70261-8. 21094089 PMC3025495

[acel70108-bib-0052] Woodward, M. 2019. “Cardiovascular Disease and the Female Disadvantage.” International Journal of Environmental Research and Public Health 16, no. 7: 1165. 10.3390/ijerph16071165. 30939754 PMC6479531

[acel70108-bib-0053] Xu, M. , T. Pirtskhalava , J. N. Farr , et al. 2018. “Senolytics Improve Physical Function and Increase Lifespan in Old Age.” Nature Medicine 24, no. 8: 1246–1256. 10.1038/s41591-018-0092-9. PMC6082705 29988130

[acel70108-bib-0054] Zhu, Y. , E. J. Doornebal , T. Pirtskhalava , et al. 2017. “New Agents That Target Senescent Cells: The Flavone, Fisetin, and the BCL‐X(L) Inhibitors, A1331852 and A1155463.” Aging (Albany NY) 9, no. 3: 955–963. 10.18632/aging.101202. 28273655 PMC5391241

[acel70108-bib-0055] Zhu, Y. , T. Tchkonia , T. Pirtskhalava , et al. 2015. “The Achilles’ Heel of Senescent Cells: From Transcriptome to Senolytic Drugs.” Aging Cell 14, no. 4: 644–658. 10.1111/acel.12344. 25754370 PMC4531078

