# Supplementary material for: Quercetin Reduces Vascular Senescence and Inflammation in Symptomatic Male but Not Female Coronary Artery Disease Patients
Source: Aging Cell. 2025 May 15;24(8):e70108. doi: 10.1111/acel.70108 (PMC12341813; doi:10.1111/acel.70108)
Supplement: Supplementary file 1 — Data S1. Supporting Information. [file ACEL-24-e70108-s001.docx]

**Supplementary Material**

**Quercetin reduces vascular senescence and inflammation in symptomatic male but not female coronary artery disease patients**

Pauline Mury PhD, Olina Dagher MD, Annik Fortier MSc, Ariel Diaz MD, Yoan Lamarche MD, PhD, Pierre-Emmanuel Noly MD, PhD, Marina Ibrahim MD, Pierre Pagé MD, Philippe Demers MD, PhD, Denis Bouchard MD, PhD, Pierre-Luc Bernier MD, PhD, Nancy Poirier MD, PhD, Emmanuel Moss MD, Nicolas Durlemann MD, Hughes Jeanmart MD, PhD, Michel Pellerin MD, PhD, Guillaume Lettre PhD, Nathalie Thorin-Trescases PhD, Michel Carrier MD, Eric Thorin PhD

Supplementary Methods 1

Study Design and Population 1

Interventions 2

Follow-up 3

Endothelium-dependent relaxant function 3

Blood proteomics analysis (Olink Explorer 384) 4

Single-nucleus suspension preparation 5

Processing of single-nucleus RNA sequencing data 6

Differential expression analysis and gene set variation analysis 7

Connectome Analysis 7

Availability of Data 9

Supplementary Tables 10

Table S1. Inclusion and exclusion criteria of the study 10

Table S2. General characteristics of the intent-to-treat cohort divided by sex and treatment groups 11

Supplementary Figures 14

Figure S1. Flow-chart of the study 14

Figure S2. Cell-type distribution divided by sex and treatment 15

Figure S3. Sex-dependent pathway enrichment analysis in EC and FIB between quercetin and placebo subgroups 16

Supplementary Methods

Study Design and Population

Q-CABG (ClinicalTrials.gov, NCT04907253) was a prospective, multi-centre, randomized, double-blind, allocation-concealed and placebo-controlled study. The trial protocol was approved by each local institutional review board and by Health Canada and was conducted in accordance with the principles of Good Clinical Practice and the trial conformed to the principles outlined in the Declaration of Helsinki. Its design and rationale have been published previously.^23^

Patients ≥18 years of age who experienced a recent acute coronary syndrome (ACS) within the previous month, including a diagnosis of unstable angina, non-ST elevation myocardial infarction (NSTEMI) or ST elevation myocardial infarction (STEMI), and who were scheduled to undergo an inpatient coronary artery bypass grafting (CABG) at the Montreal Heart Institute were approached for the study. A patient was ineligible if they required a combined procedure (*e.g*. a combined CABG and valve intervention), had documented severe kidney or liver failure, were on immunosuppressive therapy or if they were pregnant or breastfeeding. Off-pump CABG was not excluded given that surgical strategy was often unknown at the time of recruitment, however, emergent cases and those that required preoperative mechanical circulatory support at the time of recruitment were excluded. Administration of non-steroidal anti-inflammatory drugs or colchicine were not exclusion criteria. Complete inclusion and exclusion criteria are outlined in Supplementary Table 1.

Interventions

After obtaining written consent, patients were then randomly assigned in a 1:1 ratio to either the intervention arm, in which they received quercetin supplementation (500 mg *per os* bid) or the control arm, in which they received a placebo (identical looking green capsule also given *per os* bid). The randomization list was computer-generated by an unblinded biostatistician from the Montreal Health Innovations Coordinating Center (MHICC). The trial medication and matching placebo were provided free of charge by Advanced Orthomolecular Research (AOR) Inc. (Calgary, Alberta, Canada); they had no role in the design nor conduct of the trial.

Quercetin or placebo administration was started 2 days before planned surgery and was continued after surgery until hospital discharge or for a maximum of seven postoperative days. Hospital stay was not prolonged for the sole purpose of the study. All participants were managed by their own primary care team and were treated according to national guidelines that include optimal medical therapy for the treatment of acute myocardial infarctions. Being cognisant of the challenges inherent to clinical trials and the likelihood of protocol deviations, we chose to use an intention-to-treat (ITT) analysis.

Follow-up

Quercetin is a very well-tolerated substance. It is approved by the U.S. Food and Drug Administration, Health Canada and the European Medicines Agency, among many other public health agencies, and sold as an over-the-counter natural product in Canada. Regardless, tolerance of the trial therapy was still assessed on a continuous basis during patients’ hospital stay and only had to be stopped in two patients who withdrew their consent during the study. No allergic reaction nor undesirable effects were reported to be due to the trial drug.

As mentioned above, the trial therapy was stopped at hospital discharge. Participants were contacted over the phone by a member of the research team at 1-month post-surgery. Information about their post-operative recovery, including symptoms or complications (wound infection, recurrent angina, recurrent hospitalization, atrial fibrillation, stroke, etc.) was collected.

Endothelium-dependent relaxant function

Two 2-mm long segments of freshly collected ITA were mounted in a wire myograph filled by 10 mL of physiological salt solution, as previously described. We recorded isometric changes in tension: arterial segments were pre-constricted with U46619 (from 10nM to 100nM), a synthetic analogue of thromboxane A_2_; at the plateau of the constriction, segments were relaxed by cumulative addition of increasing concentrations (from 1nM to 10µM) of ACh to assess endothelium-dependent relaxation. At the end of the experiment, the segment was maximally constricted with 127mM KCl-physiological solution (NaCl replaced by KCl to induce maximal depolarization of the vascular smooth muscle cells) to calculate the percent of constriction induced by U46619. The concentration of ACh inducing 50% of relaxation (ACh-EC_50_), indicative of the vascular sensitivity to ACh, as well as the maximal relaxation (E_max_), were calculated to characterize endothelial function.

Blood proteomics analysis (Olink Explorer 384)

Proteomics data were generated using the Olink® platform at McGill Genome Center (Montreal, QC, Canada), which utilizes proximity extension assay (PEA) technology for high-throughput and sensitive biomarker detection. Olink Explore 384 Inflammation panel was used to profile protein inflammatory signature, at the peak of inflammation (POD4), in 40 randomly selected patients (30 men and 10 women) from the ITT population. Data are presented as normalized protein expression (NPX) units, which are logarithmically scaled using a log_2_ transformation.

Proteomics analyses were performed using R software (v 4.3.2). Multiple linear regression analyses were performed using Limma package. After removing non applicable values in the 384 protein expression levels, principal component analysis was performed. Then, multiple linear regression analysis was done on the total cohort, for the 4 following subsets of data: 1/ placebo patients, 2/ quercetin patients, 3/ men patients, and 4/ women patients. Sex, age, hypertension, dyslipidemia, BMI, and duration of clamping (surgery) were implemented as covariables in the model of regression. Proteins with an adjusted P-value < 0.05 (Benjamini-Hochberg correction) and log2FC > 0.25 were considered as differentially expressed protein (DEP). All DEP, in each comparison, were visualised in a Volcano plot using EnhancedVolcano R package (<https://github.com/kevinblighe/EnhancedVolcano>).

Single-nucleus suspension preparation

To evaluate the transcriptomic signature of the ITA wall, we used a snRNA-seq approach, as previously reported (5). We randomly selected 12 patients *per* protocol. This small cohort included 3 men placebo, 3 men quercetin, 3 women placebo and 3 women quercetin.

Fifty milligrams of the segment of ITA (from a segment different from that used for the assay assessing endothelial function) were used to obtain single-nuclei. Nuclei were isolated by combining enzymatic treatment with gentle mechanical dissociation using GentleMACS Octo Dissociator (Miltenyi Biotec). Briefly, frozen segments of ITA were put into the Nuclei Extraction Buffer in C-Tube and processed for 5 min. After 2 steps of washing and filtration (70 and 20 µm, respectively), nuclei were counted both automatically (Countess^TM^ II Automated Cell Counter) and manually (hemacytometer) to load 10,000 nuclei at 700-1200 nuclei/µL in a Chromium Controller instrument (10X Genomics).

Single-nucleus libraries were then prepared *per* the Next GEM Single Cell 3’ Dual Indexing kits (10X Genomics) using a Chromium Controller instrument to generate single-nucleus gel bead-in-emulsion (GEMs). Then, GEMs were reverse transcribed into cDNAs that were then amplified and cleaned up using SPRIselect Reagent Kit (Beckman Coulter). The barcoded sequencing libraries were constructed by enzymatic fragmentation, end-repair, A-tailing, adaptor ligation, ligation cleanup, sample index polymerase chain reaction (PCR), and PCR cleanup using 10X Genomics kit for sequencing in Illumina NovaSeq 6000 S4 PE100 platform.

Processing of single-nucleus RNA sequencing data

Fastq files were processed using CellRanger (10X Genomics, version 7.0.1) to decompose cell barcodes and align cDNA sequences against the human genomic reference (hg19) provided by 10X Genomics. Sample quality was assessed based on the barcode rank plot created by CellRanger. All the snRNA-seq analyses were done using R software (v 4.3.2). We performed standard pre-processing workflow (filtering out nuclei with >5% mitochondrial genes, plus keeping ones comprising between 100 and 6000 genes and less than 10,000 transcripts per nucleus), dimensionality reduction using SCTransform / uniform manifold approximation and projection (UMAP) functions (<https://github.com/satijalab/sctransform>) and clustering using Seurat on each of the 12 patients independently. Then, we filtered out potential doublets using DoubletFinder R package (<https://github.com/chris-mcginnis-ucsf/DoubletFinder>) in each Seurat object. The 12 filtered Seurat objects were then merged and integrated all together using the *IntegrateData* function from Seurat. Likely due to tissue complexity of diseased patients, we finally lost about a third of the 10,000 nuclei loaded and expected *per* patient. After all quality control filtering, we retained 79,201 nuclei in clustering with an average of 1089 genes and 2260 unique molecular identifiers (UMI) *per* cell. More nuclei were retrieved from men placebo samples than the 3 other groups (Men placebo=28,504 *vs.* Men quercetin=16,988 *vs.* Women placebo=12,346 *vs.* Women quercetin=21,363 nuclei).

Clusters were identified at resolution 0.5 using the *FindClusters* function, for the following cell-types: smooth muscle cell, endothelial cell, immune cell, fibroblast and pericyte. These cell types were automatically identified using the annotation tool Census (<https://github.com/sjdlabgroup/Census>), and manually confirmed using *DimPlot* visualization of cell type canonical markers.^20^

Differential expression analysis and gene set variation analysis

In each of the 12 samples, we added several clinical characteristics as metadata to the Seurat object, which are respectively randomisation, sex, age, EC_50_, E_max_, dyslipidemia, hypertension, BMI, diabetes, hs-CRP, and duration of clamping (surgery). Because “BMI” was different in the two populations of selected tissue samples, we used the Seurat *SCTransform* function regressing for “BMI” to preprocess our dataset. Differential expression analysis was performed using the Seurat *FindMarkers* function with default parameters. Differentially expressed genes (DEGs) between conditions with *P* values < 0.01 (Benjamini-Hochberg correction) and log2 fold change (L2FC) > 0.25 and < -0.25 were considered significant. Gene set variation analysis (GSVA) was done on Hallmark MSigDB collections using GSVA R package. The resulting out file was then added as a new assay into the Seurat object.

Connectome Analysis

Cell-cell interaction data were generated using the R software Connectome (v 1.0.1) (<https://msraredon.github.io/Connectome/>). The *DifferentialConnectome* function was used in our data to identify and highlight major perturbed cell-cell communication pathways between quercetin-placebo groups in both sexes separately. To do this, we started by calculating connectomic network for each treatment group separately, using Seurat *SplitObject* function, and then compared the two. Significant ligand-receptor interactions were visualized in two ways: 1/ Differential Scoring Plot, 2/ Circos Plot. The differential scoring is composed by 3 aligned heatmaps for ligand log fold change, receptor log fold change and score of perturbation (product of the two first plots). This allowed us to understand how quercetin treatment over- or under-expressed ligand, receptor and interactions. The Circos plot was used to plot interactions where ligand and receptors are differentially expressed in men quercetin group (*vs.* placebo group).

*Statistical Analysis*

All statistical analyses were performed using SAS Version 9.4 or higher (SAS Institute Inc., Cary, NC, USA). All statistical tests were two-tailed and a p-value < 0.05 was considered statistically significant. No adjustment for multiple testing was done and no missing data was imputed.

*Analyses of Baseline Parameters.* Continuous variables are shown as mean ± standard error of the mean (SEM) or median (25th and 75th percentiles). Groups were compared using Student’s t-test or Mann-Whitney-Wilcoxon test for continuous variables. Categorical data are presented as absolute frequencies with percentages and groups were compared using chi-square or Fisher exact test.

*Analyses of hs-CRP blood levels.* Changes from baseline to follow-up in blood levels of hs-CRP were compared between the two groups (quercetin or placebo) using a 2-way repeated measures covariance analysis (ANCOVA), including the following terms: groups (quercetin or placebo), time points (POD1, POD4, POD7), an interaction term between the groups and time points and a term for the baseline hs-CRP value at t-2 (morning before first dose). Whether the interaction term was statistically significant or not, contrasts were produced to compare groups on POD4 for the primary objective, and then for other indicated time points as exploratory analyses.

*Analyses of Olink systemic proteomic inflammatory signature.* Proteomics analyses were performed using R software (v 4.3.2). Multiple linear regression analyses were performed using Limma package (4).

*Analyses of EC_50_.* Because of their non-normal distribution, EC_50_ (the concentration of ACh that induces 50% of the maximal relaxation measured in each donor arterial segment) in placebo and quercetin groups were compared using Mann-Whitney-Wilcoxon test.

*Analysis of snRNA-seq transcriptomic*. The data set was analyzed as previously described.(5)

Availability of Data

All single-cell data for the study have been deposited in the NCBI’s Gene Expression Omnibus database (GSE278420).

Supplementary Tables

| **Table S1.** Inclusion and exclusion criteria of the study |
| --- |

| **Inclusion criteria** | **Exclusion criteria** |
| --- | --- |
| - to be able to speak French or English - to be able to give free and enlighten consent - be hospitalized and waiting for a cardiac surgery of revascularization - to have had a myocardial infarction (MI) within the past 30 days or to be in a stable angina before the surgery | - to be in a stable state without MI in the last 30 days - have a cardiac surgery concomitant to the cardiac surgery of revascularization - have an infection in the last 30 days - to have renal insufficiency (GFR less than 30) - to have a liver disease (AST, ALT or bilirubin ˃ 2X normal values) - to have a known cirrhosis - to have a history of breast cancer or other tumors estrogen-dependent - to be intolerant to flavonoids, niacine or ascorbic acid - take quinolone - need for a quinolone during post-op - not being able to give a free and enlighten consent - not being able to speak French of English - take quercetin as a supplement |

ALT, Alanine transaminase; AST, Asparate aminotransferase; GFR, Glomerular filtration rate; MI, Myocardial infarction.

| **Table S2.** General characteristics of the intent-to-treat cohort divided by sex and treatment groups at baseline (pre-treatment). | | | | | | | | | |  |
| --- | --- | --- | --- | --- | --- | --- | --- | --- | --- | --- |
|  | Men | | | Women | | | | p-value |  |  |
|  | Quercetin | | Placebo | | Quercetin | Placebo | |  |  |  |
|  | (n = 39) | (n = 39) | | (n = 8) | | | (n = 11) |  |  |  |
| **Cardiovascular risk factors** |  |  | |  | | |  |  |  |  |
| Age (years) | 68.7 ± 1.3 | 64.4 ± 1.7 | | 67.3 ± 2.5 | | | 69.8 ± 2.5 | 0.37 |  |  |
| BMI (kg/m^2^) | 29.9 ± 0.8 | 28.7 ± 0.8 | | 26.4 ± 0.5 | | | 31.7 ± 2.3 | 0.87 |  |  |
| Hypertension | 31 (79.4%) | 31 (79.4%) | | 6 (75.0%) | | | 9 (81.8%) | 1.00 |  |  |
| Dyslipidemia | 36 (92.3%) | 36 (92.3%) | | 8 (100%) | | | 10 (90.9%) | 1.00 |  |  |
| Diabetes mellitus | 17 (43.6%) | 16 (41.0%) | | 2 (25.0%) | | | 3 (27.3%) | 0.20 |  |  |
| COPD | 5 (12.8%) | 3 (7.7%) | | 1 (12.5%) | | | 2 (18.2%) | 0.45 |  |  |
| Obstructive sleep apnea | 5 (12.8%) | 7 (17.9%) | | 0 (0.0%) | | | 2 (18.2%) | 0.73 |  |  |
| Previous stroke | 2 (5.1%) | 2 (5.1%) | | 0 (0.0%) | | | 1 (9.1%) | 1.00 |  |  |
| Previous TIA | 1 (2.6%) | 1 (2.6%) | | 1 (12.5%) | | | 0 (0.0%) | 0.48 |  |  |
| Peripheral vascular disease | 7 (17.9%) | 2 (5.1%) | | 0 (0.0%) | | | 1 (9.1%) | 0.68 |  |  |
| Smoking |  |  | |  | | |  |  |  |  |
| Active | 8 (20.5%) | 8 (20.5%) | | 2 (25.0%) | | | 0 (0.0%) | 0.35 |  |  |
| Ex-smoker | 16 (41.0%) | 18 (46.1%) | | 3 (37.5%) | | | 5 (45.5%) | 1.00 |  |  |
| Previous MI | 9 (23.0%) | 7 (17.9%) | | 1 (12.5%) | | | 0 (0.0%) | 0.18 |  |  |
| Previous PCI | 9 (23.0%) | 7 (17.9%) | | 1 (12.5%) | | | 1 (9.1%) | 0.51 |  |  |
| Chronic kidney disease | 8 (20.5%) | 3 (7.7%) | | 1 (12.5%) | | | 1 (9.1%) | 1.00 |  |  |
| Known AF | 4 (10.3%) | 0 (0.0%) | | 1 (12.5%) | | | 1 (9.1%) | 0.33 |  |  |
| Alcohol abuse | 4 (10.3%) | 2 (5.1%) | | 0 (0.0%) | | | 0 (0.0%) | 0.59 |  |  |
| Illicit drug use | 1 (2.6%) | 0 (0.0%) | | 0 (0.0%) | | | 0 (0.0%) | 1.00 |  |  |
| **Home medication** |  |  | |  | | |  |  |  |  |
| Aspirin | 20 (51.3%) | 29 (74.4%) | | 4 (50.0%) | | | 7 (63.6%) | 0.69 |  |  |
| Other anti-platelet agent | 4 (10.3%) | 5 (12.8%) | | 0 (0.0%) | | | 0 (0.0%) | 0.20 |  |  |
| Warfarin | 1 (2.6%) | 0 (0.0%) | | 0 (0.0%) | | | 0 (0.0%) | 1.00 |  |  |
| DOAC | 4 (10.3%) | 0 (0.0%) | | 1 (12.5%) | | | 0 (0.0%) | 1.00 |  |  |
| Statin | 28 (71.8%) | 32 (82.1%) | | 5 (62.5%) | | | 8 (72.7%) | 0.44 |  |  |
| Beta blocker | 15 (38.5%) | 14 (35.9%) | | 4 (50.0%) | | | 4 (36.4%) | 0.69 |  |  |
| ACEI | 12 (30.8%) | 10 (25.6%) | | 1 (12.5%) | | | 4 (36.4%) | 0.87 |  |  |
| ARA II | 7 (17.9%) | 9 (23.0%) | | 2 (25.0%) | | | 3 (27.3%) | 0.58 |  |  |
| Hypoglycemic agent | 15 (38.5%) | 14 (35.9%) | | 2 (25.0%) | | | 3 (27.3%) | 0.37 |  |  |
| SGLT2i | 3 (7.7%) | 7 (17.9%) | | 0 (0.0%) | | | 0 (0.0%) | 0.20 |  |  |
| DPP4 | 2 (5.1%) | 3 (7.7%) | | 2 (25.0%) | | | 0 (0.0%) | 0.62 |  |  |
| Insulin | 2 (5.1%) | 1 (2.6%) | | 0 (0.0%) | | | 1 (9.1%) | 1.00 |  |  |
| Gabapentin | 5 (12.8%) | 1 (2.6%) | | 1 (12.5%) | | | 0 (0.0%) | 1.00 |  |  |
| HRT | 0 (0.0%) | 0 (0.0%) | | 1 (12.5%) | | | 1 (9.1%) | **0.04*** |  |  |
| NSAID | 0 (0.0%) | 0 (0.0%) | | 1 (12.5%) | | | 0 (0.0%) | 0.20 |  |  |
| **Clinical presentation** |  |  | |  | | |  |  |  |  |
| Unstable angina | 19 (48.7%) | 20 (51.3%) | | 4 (50.0%) | | | 5 (45.5%) | 0.81 |  |  |
| NSTEMI | 15 (38.5%) | 17 (43.6%) | | 4 (50.0%) | | | 5 (45.5%) |  |  |  |
| STEMI | 5 (12.8%) | 2 (5.1%) | | 0 (0.0%) | | | 1 (9.1%) |  |  |  |
| Preoperative IABP | 0 (0.0%) | 3 (7.7%) | | 0 (0.0%) | | | 0 (0.0%) | 1.00 |  |  |
| LVEF, % | 47.7 ± 2.1 | 50.9 ± 1.4 | | 53.1 ± 5.1 | | | 53.6 ± 2.0 | 0.15 |  |  |
| RV dysfunction | 6 (15.4%) | 1 (2.6%) | | 0 (0.0%) | | | 0 (0.0%) | 0.34 |  |  |
| **Biochemistry** |  |  | |  | | |  |  |  |  |
| Hs-CRP (mg/L) | 8.5 ± 6.0 | 6.5 ± 1.8 | | 3.3 ± 1.1 | | | 9.2 ± 5.0 | 0.80 |  |  |
| LDL cholesterol | 1.89 ± 0.19 | 1.93 ± 0.18 | | 2.20 ± 0.36 | | | 1.85 ± 0.23 | 0.80 |  |  |
| HDL cholesterol | 1.12 ± 0.06 | 1.18 ± 0.05 | | 1.34 ± 0.11 | | | 1.15 ± 0.11 | 0.47 |  |  |
| HbA1c, % | 6.42 ± 0.25 | 6.18 ± 0.23 | | 5.78 ± 0.22 | | | 6.21 ± 0.36 | 0.45 |  |  |
| Creatinine | 89.4 ± 3.2 | 80.5 ± 2.2 | | 69.8 ± 9.3 | | | 72.3 ± 6.3 | **0.005**** |  |  |
| Hemoglobin | 142 ± 2.1 | 138 ± 2.5 | | 126 ± 6.5 | | | 125 ± 3.6 | **<0.001**** |  |  |
| **Surgical profile** |  |  | |  | | |  |  |  |  |
| Syntax score | 21.0 ± 1.3 | 20.9 ± 1.6 | | 13.3 ± 2.1 | | | 21.4 ± 1.8 | 0.21 |  |  |
| STS score, % | 1.20 ± 0.17 | 0.84 ± 0.11 | | 1.78 ± 0.40 | | | 1.40 ± 0.24 | 0.10 |  |  |
| Euroscore II, % | 2.49 ± 0.62 | 1.19 ± 0.10 | | 2.53 ± 0.7 | | | 1.49 ± 0.1 | 0.81 |  |  |
| Off-pump | 4 (10.3%) | 9 (23.0%) | | 3 (37.5%) | | | 4 (36.4%) | 0.15 |  |  |
| CPB time (min) | 80.7 ± 4.84 | 81.0 ± 4.86 | | 78.3 ± 8.7 | | | 106 ± 13.1 | 0.24 |  |  |
| Aortic clamp duration (min) | 63.3 ± 4.33 | 63.6 ± 3.70 | | 58.5 ± 7.0 | | | 82.0 ± 8.7 | 0.40 |  |  |
| Blood cardioplegia | 12 (30.8%) | 12 (30.8%) | | 2 (25.0%) | | | 3 (27.3%) | 0.19 |  |  |
| Del Nido cardioplegia | 22 (56.4%) | 18 (46.1%) | | 3 (37.5%) | | | 4 (36.4%) |  |  |  |
| Number of distal anastomoses |  |  | |  | | |  |  |  |  |
| 1 | 2 (5.1%) | 2 (5.1%) | | 1 (12.5%) | | | 1 (9.1%) | 0.53 |  |  |
| 2 | 6 (15.4%) | 8 (20.5%) | | 1 (12.5%) | | | 1 (9.1%) |  |  |  |
| 3 | 22 (56.4%) | 15 (38.5%) | | 5 (62.5%) | | | 7 (63.6%) |  |  |  |
| 4 | 6 (15.4%) | 12 (30.8%) | | 1 (12.5%) | | | 1 (9.1%) |  |  |  |
| 5 | 3 (7.7%) | 2 (5.1%) | | 0 (0.0%) | | | 1 (9.1%) |  |  |  |
| Skeletonized ITA harvesting | 25 (64.1%) | 25 (64.1%) | | 6 (75.0%) | | | 6 (54.5%) | 0.94 |  |  |

Values are expressed as mean ± SEM, and percent within the group. *: p<0.05 Men *versus* Women (for each sex, baseline values in placebo and quercetin groups were not statistically different and were combined); statistical analyses between sexes were performed using ANOVA for continuous variables and a chi-square for categorial variables.

ACEI, Angiotensin Converting Enzyme Inhibitor; AF, atrial fibrillation; ARA, Angiotensin II receptor antagonist; BMI, body mass index; CBP, cardiopulmonary bypass; COPD, chronic obstructive pulmonary disease; DOAC, direct oral anticoagulant; DPP4, dipeptidyl peptidase 4; HbA1c, hemoglobin A1c; HRT, hormone replacement therapy; IABP, intra-aortic ballon pump; ITA, internal thoracic artery; LVEF, left ventricular ejection fraction; MI, myocardial infarction; NSAID, non-steroidal anti-inflammatory drug; NSTEMI, non-ST elevation myocardial infarction; PCI, percutaneous coronary intervention; RV, right ventricular; SGLT2i, sodium-glucose cotransporter-2 inhibitor; STEMI, ST elevation myocardial infarction; STS, society of thoracic surgeons; SV, saphenous veins; TIA, transient ischemic attack.

Supplementary Figures


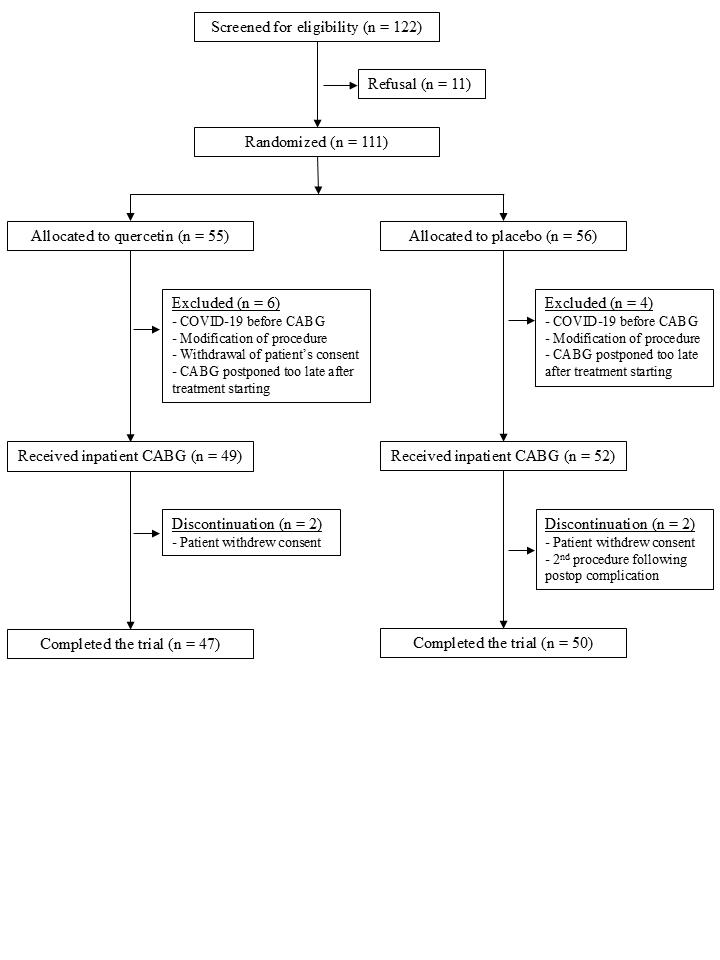


**Figure S1.** Flow-chart of the study


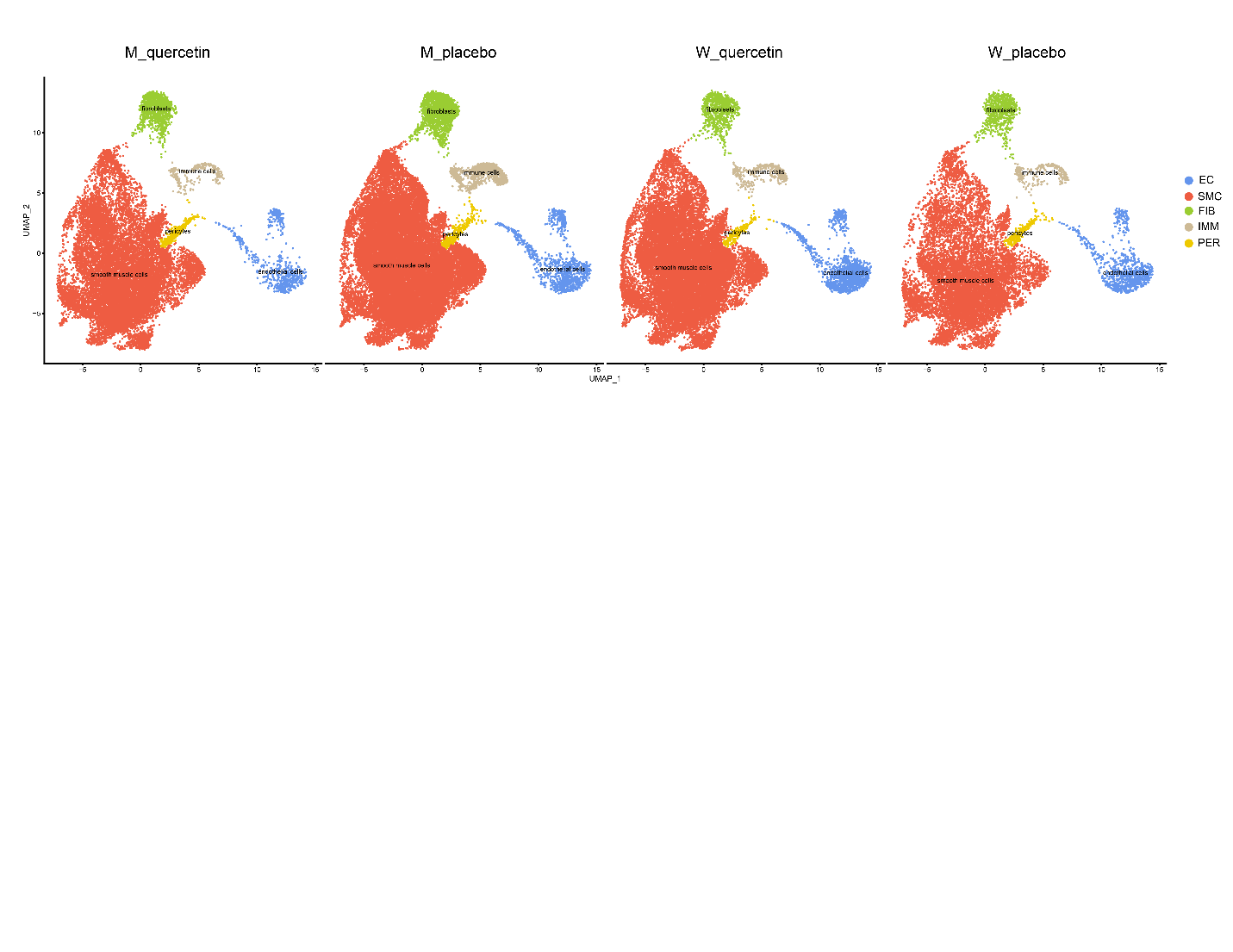


Figure S2. Cell-type distribution divided by sex and treatment**.** UMAP plot of annotated cell-types present in arteries, separated by sex and randomisation groups.

**
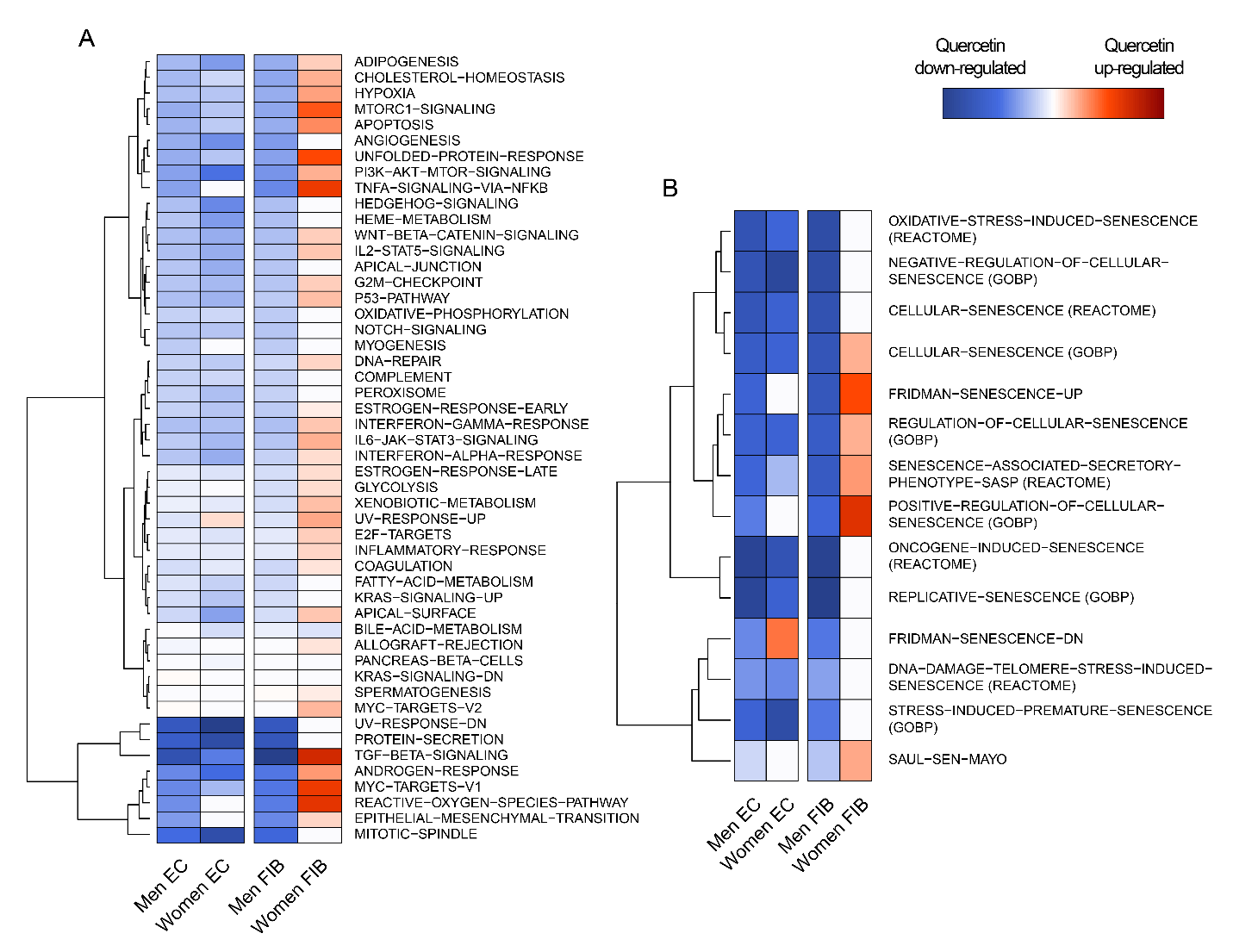
**

Figure S3. Sex-dependent pathway enrichment analysis in EC and FIB between quercetin and placebo subgroups**. A**. Heatmap columns from Figure 4 showing GSVA analysis of Hallmark collection in EC and FIB. Columns results from comparison between quercetin and placebo subgroups, in each sex independently. **B.** Heatmap columns from Figure 4 showing GSVA analysis of selected senescence-related gene sets in EC and FIB. Columns results from comparison between quercetin and placebo subgroups, in each sex independently. Red and blue squares represent overexpressed and downregulated pathways by quercetin, respectively.
